# Modeling Health Effects of Alternative Treatment Options during Surgical Delay to Inform Prioritization of Surgical Care

**DOI:** 10.1177/0272989X261445466

**Published:** 2026-05-19

**Authors:** Anouk van Alphen, Esmee Venema, Bada Yang, Vivian Reckers–Droog, Linde Huis in ‘t Veld, Rob Baatenburg de Jong, Hester Lingsma, Value Based Operation Room Triage Team Collaborators, Philip de Reuver

**Affiliations:** Department of Otorhinolaryngology, Erasmus University Medical Center, Rotterdam, The Netherlands; Department of Public Health, Erasmus University Medical Center, Rotterdam, The Netherlands; Department of Emergency Medicine, Erasmus University Medical Center, Rotterdam, The Netherlands; Julius Center for Health Sciences and Primary Care, University Medical Center Utrecht, Utrecht, The Netherlands; Department of Health Economics, Erasmus School of Health Policy & Management, Erasmus University Rotterdam, Rotterdam, The Netherlands; Julius Center for Health Sciences and Primary Care, University Medical Center Utrecht, Utrecht, The Netherlands; Department of Otorhinolaryngology, Erasmus University Medical Center, Rotterdam, The Netherlands; Department of Public Health, Erasmus University Medical Center, Rotterdam, The Netherlands

**Keywords:** surgery, decision modelling, prioritization, resources

## Abstract

**Objectives:**

To estimate the health effects of temporarily substituting surgeries with alternative treatments to inform discussions on prioritization strategies during surgical capacity constraints.

**Study Setting and Design:**

This study was conducted in the context of the Dutch health care system. We used a decision model to estimate the health effects of temporal substituting surgery with an alternative treatment option for 11 diseases. Model outcome was the number of disability-adjusted life-years (DALYs) associated with the alternative treatment during the waiting period for surgery. For each “disease–surgery” combination, the alternative treatment with the lowest DALYs was labeled the “best alternative.” These “best alternatives” were ranked in descending order of DALYs, suggesting a prioritization of surgeries.

**Data Sources and Analytic Sample:**

Parameter values were obtained from registries, scientific literature, and expert consultations.

**Principal Findings:**

A total of 23 “disease–surgery–alternative treatment” combinations were evaluated. Substituting immediate pacemaker implantation with optimal medical therapy for symptomatic bradycardia results in the largest health loss per month, estimated at 0.13 DALYs (95% confidence interval [CI] 0.08 to 0.19). Replacing implantable cardioverter–defibrillator implantation with optimal medical therapy for ventricular arrhythmias results in the second-largest health loss of 0.08 DALYs per month delay (95% CI 0.05 to 0.10). In contrast, surgeries in which substitution with alternative treatments results in minimal health impact include radiation therapy instead of laser treatment for laryngeal cancer T1–2 (−0.01 DALY, 95% CI −0.02 to 0.00) and chemoradiation instead of surgical resection for laryngeal cancer T3–4 (−0.01 DALY, 95% CI −0.02 to 0.01).

**Conclusions:**

Our study facilitates the comparison of surgery and alternative treatment options across diseases and surgical disciplines and can contribute evidence-based discussions on prioritization among health care stakeholders.

**Highlights:**

- This study quantifies the health impact of substituting surgery with alternative treatments across 11 diseases using disability-adjusted life-years (DALYs).
- Some alternative treatments resulted in minimal or no health loss, while others were associated with substantial health detriment.
- The findings support evidence-based prioritization of surgical care by identifying where substitution is most and least harmful to patient outcomes.

Over the past few decades, health care systems worldwide have been confronted with myriad challenges.^1^ There have been staff and budget shortages, and these have coincided with increased health care utilization due to emerging technologies and aging patient populations with frequent comorbidities.^2^ The coronavirus disease (COVID-19) pandemic exposed these systems’ vulnerabilities by disrupting routine care delivery.^3,4^ Increasing efficiency will not be sufficient to tackle these issues and difficult decisions regarding the allocation of resources will have to be made.^5,6^

As in other health care fields, surgery has also been afflicted by scarce resources, such as reduced operating room capacity, insufficient staffing, and shortages of intensive care unit beds for postoperative care.^4,7–9^ This scarcity results in prolonged waiting times, which can lead to increased morbidity and mortality for patients.^10–13^ One way to alleviate the pressure on surgical care is to, if only temporarily, substitute some surgeries with alternative treatments that do not compete for surgical resources. Such substitutions are not permanent, nor less costly, but serve as the best available care until surgery is available again. By this means, scarce surgical resources are reserved for surgeries with less viable alternatives.

Given the continuing shortages in health care systems,^14,15^ such substitution might become inevitable for surgical disciplines. It is widely recognized that the process of substituting routine care is extremely difficult and that adoption is often limited.^16–18^ Therefore, initiating discussions with health care stakeholders to explore such difficulties is of great importance. Ideally, stakeholders’ comprehension of the issue should be informed by research evidence. To this end, the aim of this study is to estimate the health effects of adopting alternative treatments as a way to cope with surgical delay in times of scarcity.

## Materials and Methods

This study was conducted in collaboration with the National Health Care Institute (Dutch: Zorginstituut Nederland, hereafter ZIN). ZIN is the Dutch health care rationing authority and serves as an independent advisory organization to the Ministry of Health, Welfare and Sport. ZIN’s tasks include advising policy makers on resource allocation and substitution of care.^19^

### Disease Selection

**Figure 1:**
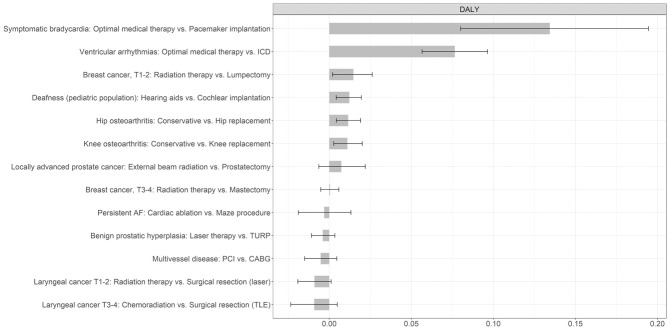
The final ranking of all “disease–surgery” combinations and their best alternative treatment. The average DALYs per month of alternative treatment are shown (*x*-axis) for all “disease–surgery–alternative” treatment combinations (*y*-axis). The estimates (gray bars) and 95% confidence intervals (black lines) are shown. AF, atrial fibrillation; CABG, coronary artery bypass graft; DALY, disability-adjusted life-year; ICD, implantable cardioverter–defibrillator; PCI, percutaneous coronary intervention; TLE, total laryngectomy; TURP, transurethral resection of the prostate.

The selection of diseases for this study was made in collaboration with ZIN, focusing on oncologic, cardiovascular, and elective surgical care. These areas had previously been identified by health care professionals, patient organizations, and ZIN as facing significant challenges in delivering appropriate care.^20^ Based on previous research,^21^ 13 diseases were selected for further investigation, and 23 disease-surgery-alternative treatment combinations were evaluated (Table 1). The alternative treatments selected for this study were made in consultation with physicians and in alignment with the existing literature, focusing on the most commonly used nonsurgical options. A detailed overview of the process of disease selection is shown in Figure 1, Supplementary Material 1.

**Table 1:** Overview of All Diseases, Including the Surgical and Alternative Treatment

	Disease	Surgery	Alternative 1	Alternative 2	Alternative 3
1	Benign prostatic hyperplasia	TURP	Conservative	Laser therapy	
2	Breast cancer, T1–2	Lumpectomy	Chemotherapy + immunotherapy	Chemotherapy	Radiation therapy
3	Breast cancer, T3–4	Mastectomy	Chemotherapy + immunotherapy	Chemotherapy	Radiation therapy
4	Deafness (pediatric population)	Cochlear implantation	Sign language	Hearing aids	
5	Hip osteoarthritis	Hip replacement	Conservative		
6	Knee osteoarthritis	Knee replacement	Conservative		
7	Laryngeal cancer T1–2	Surgical resection (laser)	Radiation therapy		
8	Laryngeal cancer T3–4	Surgical resection (TLE)	Radiation therapy	Chemoradiation	
9	Locally advanced prostate cancer	Prostatectomy	Hormone therapy	External beam radiation	
10	Multivessel disease	CABG	Optimal medical therapy	PCI	
11	Persistent AF	Maze procedure	Cardiac ablation	Optimal medical therapy	
12	Symptomatic bradycardia	Pacemaker implantation	Optimal medical therapy		
13	Ventricular arrhythmias	ICD	Optimal medical therapy		

AF, atrial fibrillation; CABG, coronary artery bypass graft; ICD, implantable cardioverter–defibrillator; PCI, percutaneous coronary intervention; TLE, total laryngectomy; TURP, transurethral resection of the prostate.

### Decision Model

The aim of the modeling was to estimate the health effects of applying an alternative treatment as a temporal substitute for surgery is a 3-state cohort state-transition model.^22^ To meet this aim, we used a previously published 3-state cohort state-transition model.^22^ This model was previously used to simulate the health effects of surgical delay, by comparing scenarios of surgical delay of 2 wk with surgical delay up to a year. In this case, no alternative treatment was administered.

In this follow-up study, we used the same model using outcomes of the alternative treatment as input parameters for the “no treatment” strategy. The 3 health states currently used were alternative treatment, surgery, and deceased. Both the alternative treatment and surgery states represent a situation after receiving treatment. The entire cohort starts in the alternative treatment state, followed by a transition to either the surgery or the deceased state. Health effects were calculated based on the duration spent in these different states. A discount rate of 1.5% was applied to these health effects, which is advised in the Dutch context.^23^ This model requires 5 input parameters: 1) the mean age of patients undergoing the surgery, 2) the survival rate of patients after the alternative treatment, 3) the survival rate of patients after the surgery, 4) the quality of life (QoL) of patients after the alternative treatment, and 5) the QoL of patients after the surgery. If either the survival rate of the alternative treatment or the surgery was lacking, the treatment effect was used to calculate the missing survival parameter. The treatment effects used were adjusted for patient population. The scenarios applied to model surgical delay (thus first receiving an alternative treatment) were created with intervals of 10 wk, starting from 2 wk up to 1 y (i.e., delay of 2 wk, 12 wk, up to 52 wk). A delay of 2 wk was considered as direct surgery. Permanent postponement was modeled as patients remaining in the alternative treatment state until they die. The cohort was simulated over a full lifespan to a maximum age of 100 y. Prioritization guided by this model was aimed at minimizing the overall societal health loss due to surgical scarcity, which accords with a utilitarian rationale. The most important assumptions in the model are detailed in Table 1, Supplementary Material 1. A description of our preliminary work can be found in our first publication on the model^22^ and the follow-up study in which we extended the model with additional surgeries.^21^

The decision model outcomes with a delay of 2 wk were compared with the outcomes at 52 wk to determine the health effect of a 50-wk delay. This was converted to disability-adjusted life-years (DALYs) per month of alternative treatment (instead of surgery), which can be interpreted as the additional health loss incurred due to the alternative treatment (i.e., surgery as reference yields zero DALYs). For each “disease–surgery–alternative treatment” combination, the model outcome was estimated. Then, for each “disease–surgery” combination, the alternative treatment that yielded the lowest DALYs was considered the best alternative since it would minimize the negative health effects relative to surgery. These best alternatives were then ranked in descending order of DALYs as a form of prioritization. Here, the logic was that surgery should be prioritized where the “disease–surgery–alternative treatment” combination had the highest DALYs, as the alternative treatment is associated with the largest health loss.

### Data Collection

Data on 4 of the input parameters (survival rate–alternative treatment, survival rate–surgery, treatment effect, and mean age) were obtained from a literature review conducted in collaboration with Cochrane Netherlands between January and April 2023. The initial focus was on using existing Cochrane Reviews. In the absence of such reviews, Epistemonikos was consulted for alternative systematic reviews.^24^ If no relevant reviews were found, primary studies were assessed. Data on mean ages were complemented with national registries. Where there was insufficient or missing data, physicians were consulted to give their considered opinion, referred to as “clinical insight.” Studies on oncologic diseases were included only if patients were eligible for surgical resection of the primary tumor. Regarding alternative treatments for oncologic surgery, neoadjuvant chemotherapy and neoadjuvant radiotherapy were excluded. If the disease severity described in the systematic review did not match our population of interest, the review was excluded. If multiple reviews were eligible per disease, the weighted mean based on both sample size and duration of follow-up was used. Details of the search strategy and review process are provided in Table 2, Supplementary Material 2.

**Table 2:** Definitive Input Parameter Values Used in the Model

Disease	Parameter	Unit	Mean	SD/Low–High	Distribution	Source
Multivessel disease: Optimal medical therapy v. CABG	Surv_altern	Probability 5-y survival	0.842	0.364005	Beta	[1]
	Surv_surg	Probability 1-y survival	0.971	0.164977	Beta	[2]
	QoL_altern	Utility	0.820	0.600 – 0.870	Triangular	Experts Delphi panels
	QoL_surg	Utility	0.970	0.780–0.980	Triangular	Experts Delphi panels
	Age	Years	66.2	57.0–75.4	Normal	NHR
Multivessel disease: PCI v. CABG	Surv_altern	Probability 1-y survival	0.977	0.148559	Beta	[2]
	Surv_surg	Probability 1-y survival	0.971	0.164977	Beta	[2]
	QoL_altern	Utility	0.970	0.870–0.980	Triangular	Experts Delphi panels
	QoL_surg	Utility	0.970	0.780–0.980	Triangular	Experts Delphi panels
	Age	Years	66.2	57.0–75.4	Normal	NHR
Deafness (pediatric population): Hearing aids v. Cochlear implantation	Surv_altern	Probability 10-year survival	1	—	N/A	Clinical insight
	Surv_surg	Probability 10-year survival	1	—	N/A	Clinical insight
	QoL_altern	Utility	0.700	0.620–0.780	Triangular	Experts Delphi panels
	QoL_surg	Utility	0.850	0.770–0.920	Triangular	Experts Delphi panels
	Age	Years	0.710	0.500–0.920	Normal	[3]
Deafness (pediatric population): Sign language v. Cochlear implantation	Surv_altern	Probability 10-year survival	1	—	N/A	Clinical insight
	Surv_surg	Probability 10-y survival	1	—	N/A	Clinical insight
	QoL_altern	Utility	0.580	0.400–0.700	Triangular	Experts Delphi panels
	QoL_surg	Utility	0.850	0.770–0.920	Triangular	Experts Delphi panels
	Age	Years	0.710	0.500–0.920	Normal	[3]
Hip osteoarthritis: Conservative v. Hip replacement	Surv_altern	Probability 5-y survival	1	—	N/A	Clinical insight
	Surv_surg	Probability 5-y survival	1	—	N/A	Clinical insight
	QoL_altern	Utility	0.710	0.600–0.810	Triangular	Experts Delphi panels
	QoL_surg	Utility	0.850	0.800–0.900	Triangular	Experts Delphi panels
	Age	Years	69.1	59.1–79.1	Normal	[4]
Ventricular arrhythmias: Optimal medical therapy v. ICD	Surv_altern	Probability 3-y survival	0.644	0.387436	Beta	[6]
	Surv_surg	Probability 4-y survival	0.819	0.271733	Beta	[5–7]
	QoL_altern	Utility	0.700	0.500–0.850	Triangular	Experts Delphi panels
	QoL_surg	Utility	0.825	0.750–0.900	Triangular	Experts Delphi panels
	Age	Years	63.0	43.0–72.0	Normal	[5–7]
Knee osteoarthritis: Conservative v. Knee replacement	Surv_altern	Probability 5-year survival	1	—	N/A	Clinical insight
	Surv_surg	Probability 5-year survival	1	—	N/A	Clinical insight
	QoL_altern	Utility	0.730	0.600–0.810	Triangular	Experts Delphi panels
	QoL_surg	Utility	0.860	0.770–0.920	Triangular	Experts Delphi panels
	Age	Years	68.0	67.9–68.1	Normal	DHD
Breast cancer, T1–2: Chemotherapy + immunotherapy v. Lumpectomy	Surv_altern	Probability 2.5-y survival	0.541	0.521–0.631	Triangular	N/A
	Surv_surg	Probability 5-y survival	0.920	0.305173	Beta	[8, 9]
	Tx_eff	HR	0.660	0.650–0.720	Lognormal	[10–14]
	QoL_altern	Utility	0.800	0.510–0.845	Triangular	Experts Delphi panels
	QoL_surg	Utility	0.850	0.670–0.900	Triangular	Experts Delphi panels
	Age	Years	55.0	53.0–59.0	Normal	[8, 9]
Breast cancer, T1–2: Chemotherapy v. Lumpectomy	Surv_altern	Probability 5-y survival	0.834	0.727–0.907	Triangular	[15–17]
	Surv_surg	Probability 5-y survival	0.920	0.305173	Beta	[8, 9]
	QoL_altern	Utility	0.675	0.500–0.780	Triangular	Experts Delphi panels
	QoL_surg	Utility	0.850	0.670–0.900	Triangular	Experts Delphi panels
	Age	Years	55.0	53.0–59.0	Normal	[8, 9]
Breast cancer, T1–2: Radiation therapy v. Lumpectomy	Surv_altern	Probability 3.5-y survival	0.913	0.193955	Beta	[18]
	Surv_surg	Probability 5-y survival	0.920	0.305173	Beta	[8, 9]
	QoL_altern	Utility	0.800	0.630–0.850	Triangular	Experts Delphi panels
	QoL_surg	Utility	0.850	0.670–0.900	Triangular	Experts Delphi panels
	Age	Years	55.0	53.0–59.0	Normal	[8, 9]
Breast cancer, T3–4: Chemotherapy + immunotherapy v. Mastectomy	Surv_altern	Probability 2.5-y survival	0.541	0.521–0.631	Triangular	NA
	Surv_surg	Probability 5-y survival	0.906	0.290560	Beta	[8, 19]
	Tx_eff	HR	0.660	0.650–0.720	Lognormal	[10–14]
	QoL_altern	Utility	0.800	0.510–0.840	Triangular	Experts Delphi panels
	QoL_surg	Utility	0.715	0.570–0.780	Triangular	Experts Delphi panels
	Age	Years	50.8	35.6–58.0	Normal	[8, 19]
Breast cancer, T3–4: Chemotherapy v. Mastectomy	Surv_altern	Probability 5-y survival	0.834	0.727–0.907	Triangular	[15–17]
	Surv_surg	Probability 5-y survival	0.906	0.290560	Beta	[8, 19]
	QoL_altern	Utility	0.675	0.500–0.780	Triangular	Experts Delphi panels
	QoL_surg	Utility	0.715	0.570–0.780	Triangular	Experts Delphi panels
	Age	Years	50.8	35.6–58.0	Normal	[8, 19]
Breast cancer, T3–4: Radiation therapy v. Mastectomy	Surv_altern	Probability 3.5-y survival	0.913	0.193955	Beta	[18]
	Surv_surg	Probability 5-y survival	0.906	0.290560	Beta	[8, 19]
	QoL_altern	Utility	0.800	0.738–0.800	Triangular	Experts Delphi panels
	QoL_surg	Utility	0.715	0.570–0.780	Triangular	Experts Delphi panels
	Age	Years	50.8	35.6–58.0	Normal	[8, 19]
Persistent AF: Cardiac ablation v. Maze procedure	Surv_altern	Probability 1-y survival	0.988	0.104772	Beta	[21, 22]
	Surv_surg	Probability 1-y survival	0.989	0.103395	Beta	[21]
	QoL_altern	Utility	0.810	0.750–0.950	Triangular	Experts Delphi panels
	QoL_surg	Utility	0.825	0.600–0.900	Triangular	Experts Delphi panels
	Age	Years	59.4	59.0–59.8	Normal	[20, 21]
Persistent AF: Optimal medical therapy v. Maze procedure	Surv_altern	Probability 5-y survival	0.939	0.206221	Beta	[23]
	Surv_surg	Probability 1-y survival	0.989	0.103395	Beta	[21]
	QoL_altern	Utility	0.780	0.600–0.850	Triangular	Experts Delphi panels
	QoL_surg	Utility	0.825	0.600–0.900	Triangular	Experts Delphi panels
	Age	Years	59.4	59.0–59.8	Normal	[20, 21]
Symptomatic bradycardia: Optimal medical therapy v. Pacemaker implantation	Surv_altern	Probability 3-y survival	0.488	0.537318	Beta	[25]
	Surv_surg	Probability 2.5-y survival	0.874	0.333893	Beta	[24]
	QoL_altern	Utility	0.675	0.300–0.850	Triangular	Experts Delphi panels
	QoL_surg	Utility	0.900	0.850–0.950	Triangular	Experts Delphi panels
	Age	Years	68.0	51.9–81.3	Normal	[24]
Locally advanced prostate cancer: External beam radiation v. Prostatectomy	Surv_altern	Probability 5-y survival	0.740	0.416817	Beta	[27]
	Surv_surg	Probability 6-y survival	0.777	0.309801	Beta	[26, 27]
	QoL_altern	Utility	0.800	0.690–0.870	Triangular	Experts Delphi panels
	QoL_surg	Utility	0.750	0.620–0.850	Triangular	Experts Delphi panels
	Age	Years	67.4	65.3–69.6	Normal	[26, 27]
Locally advanced prostate cancer: Hormone therapy v. Prostatectomy	Surv_altern	Probability 5.5-y survival	0.671	0.498912	Beta	[27–29]
	Surv_surg	Probability 6-y survival	0.777	0.309801	Beta	[26, 27]
	QoL_altern	Utility	0.800	0.690–0.850	Triangular	Experts Delphi panels
	QoL_surg	Utility	0.750	0.620–0.850	Triangular	Experts Delphi panels
	Age	Years	67.4	65.3–69.6	Normal	[26, 27]
Laryngeal cancer T1–2: Radiation therapy v. Surgical resection (laser)	Surv_altern	Probability 5-year survival	0.987	0.974–1.00	Triangular	[30]
	Surv_surg	Probability 5-y survival	0.903	0.889–0.917	Triangular	[30]
	QoL_altern	Utility	0.700	0.620–0.800	Triangular	Experts Delphi panels
	QoL_surg	Utility	0.830	0.630–0.950	Triangular	Experts Delphi panels
	Age	Years	63.0	61.4–64.6	Normal	[31]
Laryngeal cancer T3–4: Chemoradiation v. Surgical resection (TLE)	Surv_altern	Probability 5-y survival	0.435	0.498111	Beta	[32]
	Surv_surg	Probability 5-y survival	0.438	0.498389	Beta	[32, 33]
	QoL_altern	Utility	0.610	0.500–0.780	Triangular	Experts Delphi panels
	QoL_surg	Utility	0.510	0.270–0.650	Triangular	Experts Delphi panels
	Age	Years	63.0	56.0–68.6	Normal	[32, 33]
Laryngeal cancer T3–4: Radiation therapy v. Surgical resection (TLE)	Surv_altern	Probability 5-y survival	0.382	0.487052	Beta	[32]
	Surv_surg	Probability 5-y survival	0.438	0.498389	Beta	[32, 33]
	QoL_altern	Utility	0.610	0.500–0.780	Triangular	Experts Delphi panels
	QoL_surg	Utility	0.510	0.270–0.650	Triangular	Experts Delphi panels
	Age	Years	63.0	56.0–68.6	Normal	[32, 33]
Benign prostatic hyperplasia: Laser therapy v. TURP	Surv_altern	Probability 5-y survival	1	—	N/A	Clinical insight
	Surv_surg	Probability 5-y survival	1	—	N/A	Clinical insight
	QoL_altern	Utility	0.900	0.800–0.990	Triangular	Experts Delphi panels
	QoL_surg	Utility	0.850	0.800–0.900	Triangular	Experts Delphi panels
	Age	Years	71.0	70.8–71.2	Normal	DHD
Benign prostatic hyperplasia: Conservative v. TURP	Surv_altern	Probability 5-y survival	1	—	N/A	Clinical insight
	Surv_surg	Probability 5-y survival	1	—	N/A	Clinical insight
	QoL_altern	Utility	0.750	0.700–0.870	Triangular	Experts Delphi panels
	QoL_surg	Utility	0.850	0.800–0.900	Triangular	Experts Delphi panels
	Age	Years	71.0	70.8–71.2	Normal	DHD

AF, atrial fibrillation; CABG, coronary artery bypass graft; DHD, Dutch Hospital Data; HR, hazard ratio; ICD, implantable cardioverter–defibrillator; N/A, not applicable; NHR, National Heart Registry; PCI, percutaneous coronary intervention; QoL_altern, Quality of Life (QoL) of patients after the alternative treatment; QoL_surg, QoL of patients after the surgery; Surv_altern, survival probability of patients after the alternative treatment; Surv_surg, survival probability of patients after the surgery; TLE, total laryngectomy; TURP, transurethral resection of the prostate.

A full list of references used is shown in Supplementary Material 3.

Data on the remaining 2 parameters (QoL–alternative treatment and QoL–surgery) were obtained through a 2-round Delphi study involving 25 physicians, including both surgeons and nonsurgeons, representing diverse specialties (Table 4, Supplementary Material 2). A Delphi study is a series of 2 or more survey rounds interspersed with controlled feedback (meaning that participants can see the results of the previous survey round) with the aim of achieving consensus on a particular topic (ref). We made a conscious decision to recruit physicians as they are medically skilled and offer a broad perspective on a wide range of diseases and treatment options. The same method has been previously used^21,22^ and validated.^25^ Consequently, the methodological description provided here is concise, with comprehensive details of the Delphi study available in Supplementary Material 2.

First, vignettes were created for each treatment (both surgery and alternatives). These vignettes provide short descriptions of the specific health state and were derived from earlier studies.^22,25^ These descriptions were predominantly established after consultation with physicians specializing in the diseases of interest. These vignettes describe “the average patient with a certain disease,” containing descriptions of symptoms and effects on daily life. Second, the physicians were asked to value the health state described in the vignettes. To minimize the burden on the physicians, they were split into 2 panels and the vignettes equally distributed across both panels. Each physician received an invitation to participate in the Delphi study on an online Web-based Delphi tool named “Welphi.”^26^ Physicians were then redirected to Welphi, where the vignettes were presented. They were asked to estimate the QoL value of each vignette using a calibrated visual analog scale (VAS). This scale is a measure ranging from 0 (*worst imaginable health*) to 100 (*best imaginable health*).^27^ Five health-related QoL estimates (for dementia, severe depression, blindness, deafness, and infertility) from the World Health Organization Global Burden of Disease study were made available to provide reference points.^28^ Then, physicians were asked to provide a short commentary on their QoL estimate. In the second round, physicians were presented with the same vignettes and tasks but also provided with a selection of the comments, the median QoL value, and the interquartile range from the first panel round. Physicians could then alter their QoL estimate given in the first round.

### Analysis

The previously published decision model was used for model estimates.^22^ The full code of this model is available by following the GitHub link: https://github.com/bgravesteijn/Utilitarian-distribution-of-OR-capacity-during-COVID-19. A probabilistic sensitivity analysis was adopted to reflect parameter uncertainty in the results. Using 1,000 iterations, each parameter was sampled from the established distributions. The following distribution forms were used to model uncertainty: age = normal, QoL = triangular, treatment effect = lognormal, survival = triangular and beta. All analyses were performed using R open source software.^29^ For definitive input parameter values used in the model, see Table 2.

### Ethical Statement

This study was designed and executed in accordance with ethical guidelines as stated in the Helsinki Declaration and the GDPR act. Informed consent was obtained from all panel members to use and store their data for this study. No approval from an institutional review board was required because our panel members were not subject to procedures nor were they required to follow rules of behavior. As such, this study is not subject to the Medical Research Involving Human Subjects Act (WMO). This study was funded by the National Health Care Institute (Dutch: Zorginstituut Nederland as part of a Research Network HTA project, which aims to stimulate research–policy interaction. For frequently used abbreviations, see Table 3.

**Table 3:** List of Frequently Used Abbreviations in the Article

Abbreviation	Meaning
AF	Atrial fibrillation
CABG	Coronary artery bypass graft
COVID-19	Coronavirus disease 2019
DALY	Disability-adjusted life-year
DHD	Dutch Hospital data
GDPR	General Data Protection Regulation
ICD	Implantable cardioverter–defibrillator
ICU	Intensive care unit
OMT	Optimal medical therapy
PCI	Percutaneous coronary intervention
PSA	Probabilistic sensitivity analysis
QoL	Quality of life
TLE	Total laryngectomy
TURP	Transurethral resection of the prostate
VAS	Visual analogue scale
WHO	World Health Organization

### Patient and Public Involvement

Patients or the public were not involved in the design, conduct, reporting, or dissemination plans of this study.

## Results

The survival rates for the alternative treatments were mostly based on data from systematic reviews (*n* = 14), followed by clinical insight (*n* = 6), and primary study (*n* = 1). For 2 treatments, survival rates were calculated using the treatment effect. The survival rates for surgery were retrieved from systematic reviews (*n* = 17) and clinical insight (*n* = 6). Most of the mean ages came from systematic reviews (*n* = 14), followed by national registries (*n* = 5) and primary studies (*n* = 4). For 3 diseases, the population of interest was further specified based on study descriptions: laryngeal cancer T3–4 was narrowed to laryngeal cancer T4(a), symptomatic bradycardia to sick sinus syndrome or atrioventricular block, and ventricular arrhythmias to either idiopathic fibrillation or fibrillation due to myocardial infarction, coronary artery spasm, or Takotsubo disease. Three reviews—concerning persistent atrial fibrillation, breast cancer, and prostate cancer—were excluded as they involved patients with more severe disease or treatments (Supplementary Material 2). QoL weights were estimated by our 2 panels of experts. Expert panel 1 consisted of 5 surgeons and 8 nonsurgeons, while expert panel 2 included 6 surgeons and 6 nonsurgeons (Table 4, Supplementary Material 2).

### Model Outcome

Considering all 23 “disease–surgery–alternative treatment” combinations, the mean DALYs for alternative treatments was 0.05 per month of surgical delay. Breast cancer T1–2 (chemotherapy plus immunotherapy versus lumpectomy) and breast cancer T3–4 (chemotherapy plus immunotherapy versus mastectomy) yielded the largest health losses per month at 0.23 (95% 0.18–0.28) and 0.19 (95% 0.14–0.24), respectively (Table 1, Supplementary Material 3). That is, substitution in the case of these diseases leads to the greatest health losses. Conversely, the lowest DALYs were observed for laryngeal cancer T1–2 (radiation therapy versus surgical laser resection) and laryngeal cancer T3–4 (chemoradiation versus surgical resection) at −0.01 (95%: −0.02 to 0.00) and −0.01 (95%: −0.02 to 0.01), respectively (Table 1, Supplementary Material 3). The fact that the DALYs are negative implies that the nonsurgical alternative treatments even result in health gains, although this is marginal and uncertain since the confidence intervals (CIs) contain zero.

### Ranking

The best alternative treatments for each disease are illustrated in Figure 1. Substituting immediate pacemaker implantation with optimal medical therapy for symptomatic bradycardia results in the largest health loss per month, estimated at 0.13 DALYs (95% CI 0.08–0.19). Replacing implantable cardioverter–defibrillator (ICD) implantation with optimal medical therapy for ventricular arrhythmias results in the second-largest health loss of 0.08 DALYs per month delay (95% CI 0.06–0.10). At the other end of the ranking, laryngeal cancer T3–4 (chemoradiation versus surgical resection) had the lowest health losses (−0.01: 95% CI −0.02 to 0.00), followed by laryngeal cancer T1–2 (radiation therapy versus surgical laser resection) (−0.01: 95% CI −0.02 to 0.00), indicating that delaying these surgeries while providing alternative treatments does not result in health loss.

## Discussion

This study estimated the health effects of (temporarily) substituting surgery with alternative treatments. These results enable a transparent and objective comparison among alternative treatments for various diseases and should be used to inform discussions on prioritization strategies. The observed health effects of substituting surgery varied, ranging from health gains to health losses. To develop a comprehensive ranking useful for prioritization purposes, additional diseases and resources required should be included in the analysis.

Our model’s analysis of the best alternatives indicated that of the 13 disease–surgery–alternative treatment options evaluated, in 6 cases applying alternative treatment instead of immediate surgery (46%) would lead to adverse health effects. For elective surgery to treat hip and knee osteoarthritis, and for hearing loss, these findings align with the general consensus that the alternative treatments (i.e., physiotherapy or hearing aids) are suboptimal.^30,31^ Such approaches are deemed inappropriate, especially in severe manifestations of these diseases. The same is true for the cardiovascular diseases (i.e., symptomatic bradycardia and ventricular arrhythmias) where there is abundant literature supporting the positive effects of pacemaker or ICD implantation.^32,33^

Ambiguity on the health effects of substitute treatments were also observed. Seven (54%) of the disease treatments modeled had confidence intervals that included zero. This suggests some uncertainty as to whether the alternative treatment might be equivalent or even superior to surgery. It is noteworthy that this ambiguity aligns with previously published studies evaluating such surgeries. Examples include the long-standing debate on the optimal treatment strategy for treating multivessel diseases, with CABG or with PCI.^34–36^ Similarly, in the case of laryngeal cancer, it is generally acknowledged that outcomes are similar between surgical and nonsurgical treatment.^37^ Shared decision making is deemed essential in this regard. For example, for benign prostatic hyperplasia, TURP has long been considered the gold standard, but a laser procedure has now become a viable alternative. Recent studies have compared both options and reported similar improvements in urinary tract symptoms and QoL.^38,39^ The conclusion was that both treatment strategies could be recommended as they are clinically effective but that the long-term benefits were uncertain.

Our current findings extend earlier work that focused on surgical delay but did not consider alternative treatment options.^21,22,40^ In those studies, the “alternative” was no treatment at all. Since an alternative treatment was modeled in the current study, we would expect the health loss not to exceed that calculated in our previous research. This holds true for most of the diseases, for which the results are largely consistent with this argument. However, when considering the alternative to pacemaker implantation for symptomatic bradycardia, the alternative treatment appears to be worse than no treatment at all. This discrepancy was caused by a different survival rate of the alternative modeled. Rates of 0.83 and 0.79 for no treatment and optimal medical therapy, respectively, suggest that optimal medical therapy leads to a worse survival than no treatment does. This discrepancy arises from the selection of different patient populations from studies. For the no-treatment group, accurate data were lacking, and therefore, the treatment effect of ICD implantation was used.^41^ The rate of optimal medical therapy was derived from a study evaluating patients with second-degree atrioventricular block,^42^ who have more severe disease.

Substituting surgical care with alternative treatments is a complex and sensitive topic, characterized by numerous nuances on both the individual patient, physician, organizational, and national levels. Foremost, one should recognize that surgeons will not perform any surgery unless their clinical judgment deems it necessary. Therefore, we should refrain from engaging in discussions about individual decision making as such prioritization decisions will be influenced by a patient’s characteristics and specific cases might not even be eligible for alternative treatments. As such, the ranking established by our model should serve as a decision aid that structures and communicates scientific evidence to support national-level discussions (i.e., a conceptual use). It could help stakeholders think differently about prioritization issues and help in shaping legitimate and accountable policies. Nevertheless, we are well aware that also at the national level, our findings cannot be directly translated into new policies as numerous nonrational factors (e.g., personal preferences, degree of emotion involved) influence health care policy decision making.^43^ Moreover, the distribution of health effects (equity) is not addressed by the model, nor are any cost considerations, as the primary constraint in this context is personnel availability rather than financial resources. Hence, we perceive this study as an important first step in guiding discussions rather than a direct, tangible blueprint for policy development.

Our study has some limitations. To start with, most are a consequence of the model assumptions as provided in Supplementary Material 1. First, it was assumed that each treatment could be provided at any time without considering the likely clinical duration of a treatment. Similarly, the recovery period after a treatment—which could temporarily reduce QoL—was not considered. In essence, the current model greatly simplifies daily clinical practice, which reinforces the notion that the findings should not be used to directly dictate decision making.

Furthermore, one could argue that the vignettes used might not accurately capture the psychological and social dimensions of a disease, which could affect health state valuations.

Second, during data collection, it was found that the selection of diseases and treatment options did not always correspond with the literature. Although our selection was established after consultations with physicians, the disease and treatment options chosen were, in some cases, too broad. The systematic reviews used specific inclusion and exclusion criteria, resulting in specific patient populations. We further specified the patient population of interest for several diseases to mitigate this. As a result, not all diseases could be included as comprehensively as intended, and some of the obtained data ultimately proved not useful. Further extensions of our current work should pay attention to carefully selecting treatment strategies to compare.

Third, for each surgery, 1 physician from our own hospital was consulted to provide alternative treatment options. This was for pragmatic reasons but results in a somewhat academic perspective on possible treatments. In any further national policy considerations, it would be advisable to form physician panels stemming from various institutions to improve the representability of any recommendations made.

Despite these limitations, our findings have 2 implications for future thinking about prioritizing surgery. First, we argue that it is crucial to discuss our findings with health care stakeholders (e.g., physicians, model developers, and policy makers) to evaluate whether it is feasible to use the current results to inform policy making. Since prioritizing surgical care is becoming an essential endeavor both now and in the foreseeable future, discussions on what to base such decisions on are inevitable. Second, for research purposes, it would be informative to explore citizens’ perspectives on substitution and prioritization supported by our decision model to legitimize any policy making based on these.

## Conclusions

Our model outcome enables a pragmatic, evidence-based, and interdisciplinary comparison of alternative treatment options to surgery. This provides a foundation for well-informed discussions and can help health care stakeholders to critically review current prioritization practice. This is of great relevance since the current temporary substitution of surgical care might become a permanent reality in the foreseeable future due to ongoing scarcity in the health care system.

## Supplemental Material

sj-docx-1-mdm-10.1177_0272989X261445466 – Supplemental material for Modeling Health Effects of Alternative Treatment Options during Surgical Delay to Inform Prioritization of Surgical Care Supplemental material, sj-docx-1-mdm-10.1177_0272989X261445466 for Modeling Health Effects of Alternative Treatment Options during Surgical Delay to Inform Prioritization of Surgical Care by Anouk van Alphen, Esmee Venema, Bada Yang, Vivian Reckers–Droog, Linde Huis in ‘t Veld, Rob Baatenburg de Jong and Hester Lingsma in Medical Decision Making
