# Supplementary material for: Modeling Health Effects of Alternative Treatment Options during Surgical Delay to Inform Prioritization of Surgical Care
Source: Med Decis Making. 2026 May 19;46(5):661–72. doi: 10.1177/0272989X261445466 (PMC13242533; doi:10.1177/0272989X261445466)
Supplement: sj-docx-1-mdm-10.1177_0272989X261445466 – Supplemental material for Modeling Health Effects of Alternative Treatment Options during Surgical Delay to Inform Prioritization of Surgical Care [file sj-docx-1-mdm-10.1177_0272989X261445466.docx]

## Supplementary material 1

## Figure 1. An overview of the study procedure


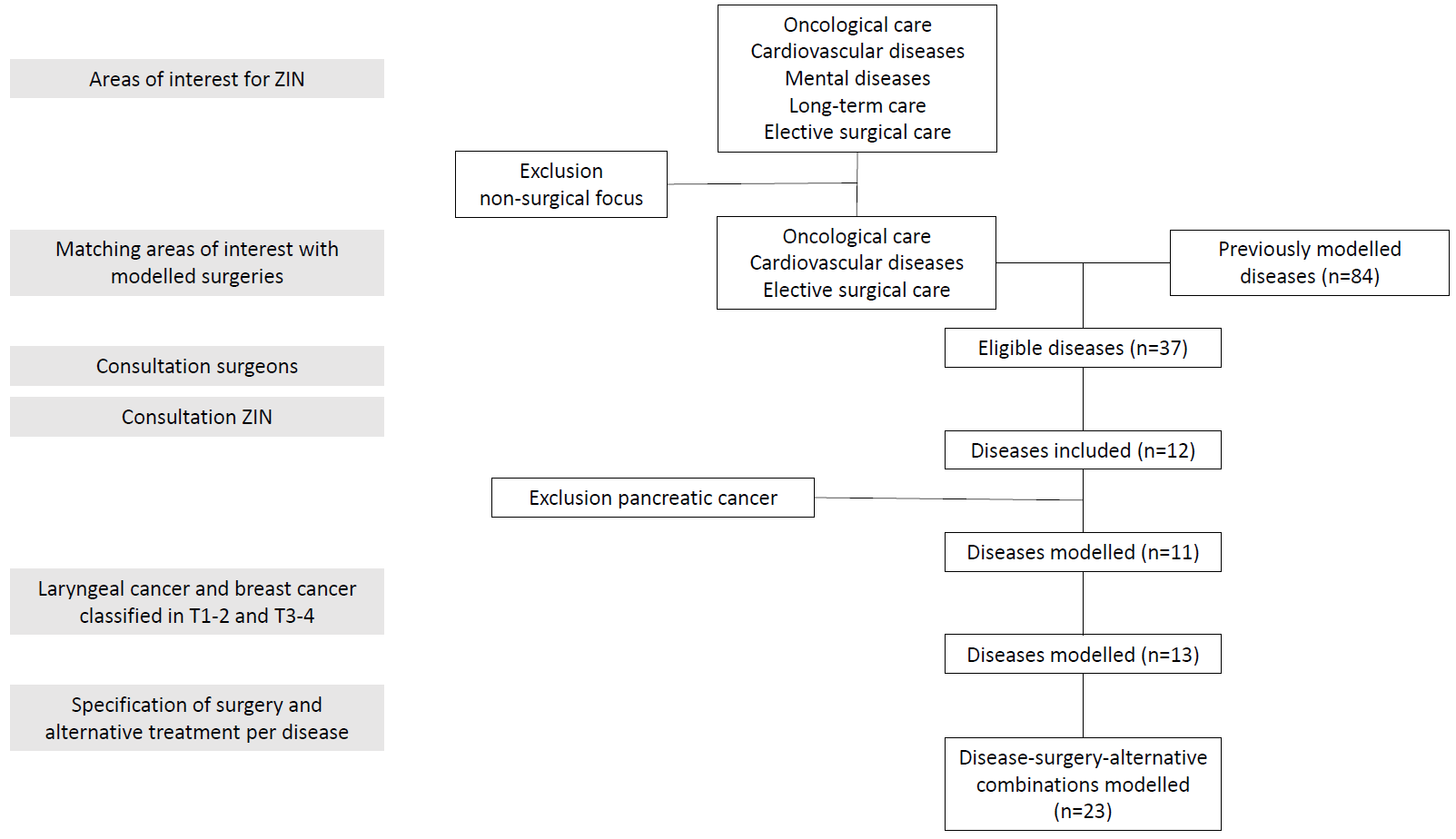


ZIN: Zorginstituut, National Health Care Institute

ZIN identified four key areas: oncological care, cardiovascular diseases, mental diseases, and long-term care. We excluded mental diseases and long-term care to focus on surgical care. In addition, ZIN prioritized elective surgical care due to its postponement risk. These criteria (oncological, cardiovascular, or elective care) guided our selection from a previous study where we modeled the health effects of surgical delays for 84 diseases. Of these (84 diseases), 37 diseases aligned with the areas of interest of our new study. For each of these diseases, the commonly performed surgeries were established. Surgeons at Erasmus University Medical Center suggested alternative treatments. For feasibility, we focused on 12 diseases, later excluding pancreatic cancer as it still involved surgery. Laryngeal and breast cancers were categorized by tumor stages T1-2 and T3-4, resulting in 13 specified diseases for surgery and alternative treatments.

## An overview of the model structure, the input parameters, and assumptions.

Figure 2. A three–state cohort state–transition model was previously developed and used to simulate the effect on health effect due to replacing a surgery with an alternative treatment.


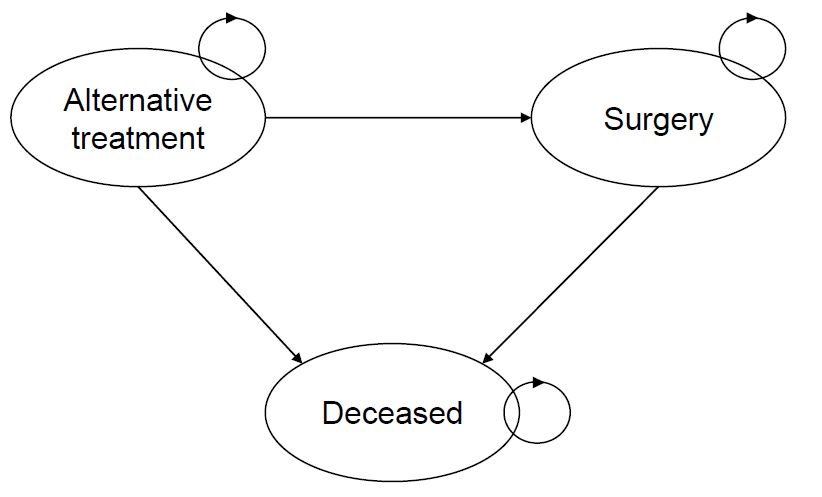


Table 1. The model has a set of underlying assumptions and limitations, each of which carries potential implications.

| **Assumption** | **Potential impact** |
| --- | --- |
| The alternative treatment is always available. There is no delay or barrier to accessing these treatments. | The model may not reflect real-world limitations or waiting times for alternative treatments, potentially overestimating their effectiveness. |
| The clinically relevant duration of an alternative treatment (i.e., chemotherapy for only several weeks) is not taken into account. | The model may not accurately reflect the time frames required for these treatments to take effect. This could lead to underestimation of their benefits if shorter durations are assumed. |
| The population described in studies on the surgical option is equivalent to the population evaluated for the alternative treatment, and vice versa. | Some of the studies used for input parameters might represent a population which is not truly equivalent to that for the other treatment option. Certain parameters could therefore overestimate or underestimate the effect. This emphasizes the need for careful consideration when generalizing the results. |
| Patient characteristics that might influence the treatment effect (e.g. comorbidities) are not included. | This might result in an oversimplified model. Patients with different profiles may have varying responses to treatments. |
| All surgeries are successful (e.g., repeat surgery is not considered). We do not include major surgical complications (serious bleeding etc.) because we assume that these complications are rare. | This could lead to an overestimate of the benefits of surgery by not accurately reflecting the real-world clinical setting. |
| Notional artificial triangular distributions are used for parameter distribution where the real distribution is not known. | By using triangular distributions, the 95% confidence intervals may not reflect the true distribution of the data. |
| The disutility of recovery time is not modelled. | This will underestimate the potential burden on patients during the post-operative period. This can impact the quality of life estimates. |
| The impact of delay on quality of life or survival is not modelled. | This may lead to an incomplete representation of the consequences of delayed treatment, especially for time-sensitive conditions. |
| Patients are assumed to have the potential for both treatments. Individual patient characteristics, such as comorbidities, which might influence the choice of treatment strategy are not considered. | This is an oversimplification of clinical decision-making. The actual choice of treatment strategy is more complex and considers patient-specific factors. |
| Patients can have the surgery at any time, including after the alternative treatment has been given. | The model's outcomes may not align with real-world scenarios. The model assumes that a surgery has a consistent level of effectiveness whenever it is given, and we know from clinical practice that certain surgeries can be less effective if delayed. |

## Supplementary material 2

## Methods and results of the literature review

A literature review was conducted between 9 January and 26 April 2023 to synthesize data on four parameters: *survival rate of alternative treatment*, *survival rate after surgery*, *treatment effect*, and *mean age*.

The review was performed using a phased approach: for a given clinical question (Patient–Intervention–Control–Outcome combination, or PICO), initially searches were performed for pre–existing Cochrane Reviews. If none were available, other systematic reviews were searched for and if no systematic reviews were available, we looked for primary studies.

Studies on cancer were only included if patients were eligible for surgical resection of the primary tumour. That is, patients with recurrent cancers were excluded. In terms of interventions, we excluded neoadjuvant chemotherapy and neoadjuvant radiotherapy. The desired outcome variable was overall survival. We also included reviews and studies that reported other survival outcomes (e.g. disease-specific survival, progression-free survival) or composite outcomes including survival (such as Major Adverse Cardiac Events).

The *Cochrane Database of Systematic Reviews* for Cochrane Reviews and Epistemonikos ([www.epistemonikos.org](http://www.epistemonikos.org)) were searched for other systematic reviews and primary studies. Our search strategies are outlined below.

A single reviewer assessed the eligibility of reviews/studies against the PICOs, and a senior reviewer was consulted when in doubt. Data on parameter estimates were extracted by one reviewer and checked by a second. The mean age of the population for a given PICO was extracted as either the mean or median age, and only included reviews or studies where the survival rate closely matched the median survival rate across all reviews on the given PICO. We did not take account of the possibility of overlapping primary studies (i.e., the same primary study being included in multiple reviews) as it was not feasible to assess the extent of any overlap.

The *survival rate* parameter was summarized as proportions and the *treatment effect* parameter as either a hazard ratio, risk ratio, or risk difference. The median and range of parameter point estimates were calculated across reviews when multiple reviews were available on a given PICO. No meta–analyses were performed.

A total of 208 unique hits were found for the five PICOs related to breast cancer and included 14 systematic reviews (9 Cochrane and 5 non-Cochrane) that covered 224 studies and 301,537 participants. For the eight PICOs relating to laryngeal or prostate cancer, we screened 397 unique hits and included 7 systematic reviews (1 Cochrane and 6 non-Cochrane) and 1 primary study. The seven reviews included 91 studies and, with the additional primary study, included a total of 45,806 participants. The search related to the six cardiology PICOs resulted in 319 unique hits, of which 8 systematic reviews (3 Cochrane and 5 non-Cochrane) met the criteria for inclusion. These 8 reviews covered 229 studies and 40,710 participants.

All reviews underwent further evaluation to establish per review the population of interest. Subsequently, three reviews were excluded due to discrepancies in the patient population studied compared to our population of interest (see Table 1 ). Following this, both sample size and survivors were determined. For parameters exhibiting a beta distribution, the alpha and beta were computed using the mean and standard deviation. The following formula was used to calculate the standard deviation:


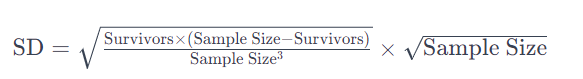


Source: [Confidence interval for a proportion | Sample Size Calculators (sample-size.net)](https://sample-size.net/confidence-interval-proportion/)

If multiple reviews were eligible per disease, the mean was weighted based on both sample size and follow-up (see Table 2).

**Table 1. Parameter data obtained after literature review**

| **Disease** | **Population of interest** | **Intervention** | **Population included** | **Sample** | **Survivors** | **Unit** | **Mean** | **SEM** | **SD** | **Reference** |
| --- | --- | --- | --- | --- | --- | --- | --- | --- | --- | --- |
| Multivessel disease | Patients with multivessel disease (eligible for CABG) | CABG (without valve surgery) | Multivessel disease, CABG | 1472 | 1430 | Prob. 1-year survival | 0.971 | 0.0043 | 0.165 | Bakhai et al. |
|  |  | PCI | Multivessel disease, PCI/PTCA | 1451 | 1418 | Prob. 1-year survival | 0.977 | 0.0039 | 0.149 | Bakhai et al. |
| Laryngeal cancer | Patients with T1-T2, M0 laryngeal cancer | Surgical resection (laser) | Laryngeal cancer T1 and T2 | 76 |  | Prob. 5-year survival | 0.987 |  |  | Warner et al. |
|  |  | Radiation therapy | Laryngeal cancer T1 and T2 | 129 |  | Prob. 5-year survival | 0.903 |  |  | Warner et al. |
|  | Patients with locally advanced resectable T3-T4, M0 laryngeal cancer | (Chemo)radiation | Laryngeal cancer T4a | 312 | 144 | Prob. 5-year survival | 0.435 | 0.0282 | 0.498 | Francis et al. |
|  |  | Surgical resection (TLE) | Laryngeal cancer T4 | 102 | 32 | Prob. 5-year survival | 0.349 | 0.0459 | 0.464 | Fu et al. |
|  |  |  | Laryngeal cancer T4a | 402 | 194 | Prob. 5-year survival | 0.460 | 0.0249 | 0.499 | Francis et al. |
| Symptomatic bradycardia | Patients with symptomatic bradycardia | Pacemaker implantation | Sick sinus syndrome or atrioventricular block | 2411 | 2107 | Prob. 2.5-year survival | 0.874 | 0.0068 | 0.334 | Dretzke et al. |
| Ventricular arrhythmias | Ventricular arrhythmias | ICD | Idiopathic ventricular fibrillation | 281 | 278 | Prob. 5-year survival | 0.990 | 0.0061 | 0.102 | Ozaydin et al. |
|  |  |  | Secondary prevention | 963 | 821 | Prob. 3-year survival | 0.755 | 0.0114 | 0.354 | Parkes et al. |
|  |  |  | MI, CAS, Takotsubo, various | 2570 | 2416 | Prob. 4-year survival | 0.824 | 0.0047 | 0.238 | van der Lingen et al. |
|  |  | Optimal medical therapy | Amiodarone use | 1060 | 867 | Prob. 3-year survival | 0.644 | 0.0119 | 0.387 | Parkes et al. |
| Persistent AF | Persistent/permanent AF | Cardiac ablation | Atrial fibrillation | 124 | 123 | Prob. 1-year survival | 0.992 | 0.008 | 0.089 | Nyong et al. |
|  |  |  | Atrial fibrillation | 3264 | 3226 | Prob. 1-year survival | 0.988 | 0.0019 | 0.109 | Berger et al. |
|  |  | Maze procedure | Persistent atrial fibrillation | 464 | 459 | Prob. 1-year survival | 0.989 | 0.0048 | 0.103 | Berger et al. |
|  |  |  | * |  |  |  |  |  |  | Barnett et al. |
|  |  | Optimal medical therapy | Amiodarone | 6291 | 6000 | Prob. 5-year survival | 0.939 | 0.0026 | 0.206 | Doyle et al. |
| Breast cancer | Patients eligible for resection (usually T3 and T4b, N0-1, M0) | Mastectomy | Modified radical mastectomy | 350 | 312 | Prob. 5-year survival | 0.891 | 0.0166 | 0.311 | Mota et al. |
|  |  |  | Mastectomy without reconstruction | 182 | 179 | Prob. 5-year survival | 0.979 |  |  | Nanda et al. |
|  |  |  | Mastectomy with reconstruction | 2845 | 2570 | Prob. 5-year survival | 0.903 |  |  | Nanda et al. |
|  |  | Radiation therapy (adjuvant) | Whole breast radiotherapy | 6531 | 6281 | Prob. 3.5-year survival | 0.913 | 0.0024 | 0.194 | Hickey et al. |
|  |  | Chemotherapy (adjuvant) | Chemotherapy with taxanes |  |  | Prob. 5-year survival | 0.844 |  |  | Willson et al. |
|  |  |  | Chemotherapy without taxanes |  |  | Prob. 5-year survival | 0.824 |  |  | Willson et al. |
|  |  |  | Ovarian function suppression with chemotherapy |  |  | Prob. 10-year survival | 0.727 |  |  | Bui et al. |
|  |  |  | Ovarian function suppression with chemotherapy |  |  | Prob. 10-year survival | 0.690 |  |  | Bui et al. |
|  |  |  | Capecitabine-containing regimens (adjuvant) |  |  | Prob. 5-year survival | 0.907 |  |  | Hoon et al. |
|  |  |  | ** |  |  |  |  |  |  | Farquhar et al. |
| Breast cancer | Patients eligible for resection (T1-T4, N0-1, M0) | Chemotherapy + immunotherapy (neoadjuvant) | Early triple-negative breast cancer |  |  | Hazard ratio | 0.660 |  |  | Li et al. |
|  |  |  | Early stage triple-negative breast cancer |  |  | Hazard ratio | 0.560 |  |  | Mittal et al. |
|  |  |  | Early stage triple-negative breast cancer patients |  |  | Hazard ratio | 0.660 |  |  | Sternschuss et al. |
|  |  |  | Early triple-negative breast cancer confirmed by immunohistochemistry |  |  | Hazard ratio | 0.650 |  |  | Xin et al. |
|  |  |  | Patients with confirmed triple negative breast cancer based on pathology |  |  | Hazard ratio | 0.720 |  |  | Zhang et al. |
|  | Patients eligible for breast-saving surgery (usually T1-2, M0) | Lumpectomy | Breast conserving with radiotherapy versus breast conserving with boost radiotherapy | 3160 | 2609 | Prob. 5-year survival | 0.924 | 0.0067 | 0.377 | Kindts et al. |
|  |  |  | Standard breast conserving | 6531 | 6072 | Prob. 5-year survival | 0.9176 | 0.0075 | 0.606 | Nanda et al. |
| Prostate cancer | Locally advanced prostate cancer, M0 | Prostatectomy | Ct3, 4, TXN | 2313 | 2018 | Prob. 5-year survival | 0.835 | 0.0069 | 0.332 | Fahmy et al. |
|  |  | External beam radiation | Ct3, 4, TXN | 201 | 156 | Prob. 5-year survival | 0.74 | 0.0294 | 0.417 | Fahmy et al. |
|  |  |  | *** |  |  |  |  |  |  | Dayes et al. |
|  |  | Hormone therapy | Locally advanced prostate cancer | 1172 | 324 | Prob. 8-year survival | 0.645904 | 0.0131 | 0.448 | Lei et al. |
|  |  |  | Locally advanced prostate cancer | 1321 | 761 | Prob. 3.5-year survival | 0.576079 | 0.0136 | 0.494 | Dong et al. |
|  |  |  | Ct3, 4, TXN | 580 | 284 | Prob. 5-year survival | 0.475 | 0.0208 | 0.501 | Fahmy et al. |

* Excluded for further analysis. Patients with biatrial Maze included, this is more extensive surgery than mini-Maze.

** Excluded for further analysis. Patients with early poor prognosis breast cancer with multiple axillary lymph node involvement.

*** Excluded for further analysis. Patients with high-risk disease.

AF: atrial fibrillation, CABG: coronary artery bypass graft, ICD: implantable cardioverter–defibrillator, PCI: percutaneous coronary intervention, TURP: transurethral resection of the prostate, CAS: coronary artery spasm, IVF: idiopathic ventricular fibrillation, MI: myocardial infarction, PCI: percutaneous coronary intervention, Prob.: probability, PTCA: percutaneous transluminal coronary angioplasty, RFCA: radiofrequency catheter ablation, SD: standard deviation, SEM: standard error of mean, TLE: total laryngectomy.

References:

Bakhai, A., et al. (2005). Percutaneous transluminal coronary angioplasty with stents versus coronary artery bypass grafting for people with stable angina or acute coronary syndromes. Cochrane Database of Systematic Reviews, 2005(1), CD004588.

Barnett, S. D., & Ad, N. (2006). Surgical ablation as treatment for the elimination of atrial fibrillation: A meta-analysis. Journal of Thoracic and Cardiovascular Surgery, 131(5), 1029–1035.

Berger, W. R., et al. (2019). Persistent atrial fibrillation: A systematic review and meta-analysis of invasive strategies. International Journal of Cardiology, 278, 137–143.

Bruijnzeel, H., et al. (2017). Evaluation of pediatric cochlear implant care throughout Europe: Is European pediatric cochlear implant care performed according to guidelines? Cochlear Implants International, 18(6), 287–296.

Bui, K. T., et al. (2020). Ovarian suppression for adjuvant treatment of hormone receptor-positive early breast cancer. Cochrane Database of Systematic Reviews, 2020(3), CD013538.

Doyle, J. F., & Ho, K. M. (2009). Benefits and risks of long-term amiodarone therapy for persistent atrial fibrillation: A meta-analysis. Mayo Clinic Proceedings, 84(3), 234–242.

Dretzke, J., et al. (2004). Dual chamber versus single chamber ventricular pacemakers for sick sinus syndrome and atrioventricular block. Cochrane Database of Systematic Reviews, 2004(2), CD003710.

Dong, Z., et al. (2015). Intermittent hormone therapy versus continuous hormone therapy for locally advanced prostate cancer: A meta-analysis. Aging Male, 18(4), 233–237.

Fahmy, O., et al. (2017). The role of radical prostatectomy and radiotherapy in treatment of locally advanced prostate cancer: A systematic review and meta-analysis. Urologia Internationalis, 99(3), 249–256.

Francis, E., et al. (2014). T4a laryngeal cancer survival: Retrospective institutional analysis and systematic review. Laryngoscope, 124(7), 1618–1623.

Fu, X., Zhou, Q., & Zhang, X. (2016). Efficacy comparison between total laryngectomy and nonsurgical organ-preservation modalities in treatment of advanced stage laryngeal cancer: A meta-analysis. Medicine (Baltimore), 95(14), e3142.

Hickey, B. E., & Lehman, M. (2021). Partial breast irradiation versus whole breast radiotherapy for early breast cancer. Cochrane Database of Systematic Reviews, 2021(8), CD007077.

Hoon, S. N., et al. (2021). Capecitabine for hormone receptor-positive versus hormone receptor-negative breast cancer. Cochrane Database of Systematic Reviews, 2021(5), CD011220.

Kindts, I., et al. (2017). Tumour bed boost radiotherapy for women after breast-conserving surgery. Cochrane Database of Systematic Reviews, 2017(11), CD011987.

Klop, C., et al. (2012). COX-2-selective NSAIDs and risk of hip or knee replacements: A population-based case-control study. Calcified Tissue International, 91(6), 387–394.

Lei, J. H., et al. (2016). Androgen-deprivation therapy alone versus combined with radiation therapy or chemotherapy for nonlocalized prostate cancer: A systematic review and meta-analysis. Asian Journal of Andrology, 18(1), 102–107.

Li, Y., et al. (2021). Efficacy and safety of adding immune checkpoint inhibitors to neoadjuvant chemotherapy against triple-negative breast cancer: A meta-analysis of randomized controlled trials. Frontiers in Oncology, 11, 657634.

Marra, G., et al. (2020). Management of patients with node-positive prostate cancer at radical prostatectomy and pelvic lymph node dissection: A systematic review. European Urology Oncology, 3(5), 565–581.

Mittal, N., et al. (2022). Immune checkpoint inhibitors as neoadjuvant therapy in early triple-negative breast cancer: A systematic review and meta-analysis. Journal of Cancer Research and Therapeutics, 18(6), 1754–1765.

Mota, B. S., et al. (2016). Nipple- and areola-sparing mastectomy for the treatment of breast cancer. Cochrane Database of Systematic Reviews, 2016(11), CD008932.

Nanda, A., et al. (2021). Oncoplastic breast-conserving surgery for women with primary breast cancer. Cochrane Database of Systematic Reviews, 2021(10), CD013658.

Nyong, J., et al. (2016). Efficacy and safety of ablation for people with non-paroxysmal atrial fibrillation. Cochrane Database of Systematic Reviews, 2016(11), CD012088.

Ozaydin, M., et al. (2015). Long-term outcome of patients with idiopathic ventricular fibrillation: A meta-analysis. Journal of Cardiovascular Electrophysiology, 26(10), 1095–1104.

Parkes, J., Bryant, J., & Milne, R. (2002). Implantable cardioverter-defibrillators in arrhythmias: A rapid and systematic review of effectiveness. Heart, 87(5), 438–442.

Pedregal-Mallo, D., et al. (2018). Oncological and functional outcomes of transoral laser surgery for laryngeal carcinoma. European Archives of Oto-Rhino-Laryngology, 275(8), 2071–2077.

Shaw, D. B., et al. (1985). Survival in second degree atrioventricular block. British Heart Journal, 53(6), 587–593.

Sternschuss, M., et al. (2021). Efficacy and safety of neoadjuvant immune checkpoint inhibitors in early-stage triple-negative breast cancer: A systematic review and meta-analysis. Journal of Cancer Research and Clinical Oncology, 147(11), 3369–3379.

van der Lingen, A. C. J., et al. (2022). Recurrent ventricular arrhythmias and mortality in cardiac arrest survivors with a reversible cause with and without an implantable cardioverter defibrillator: A systematic review. Resuscitation, 173, 76–90.

Warner, L., et al. (2014). Radiotherapy versus open surgery versus endolaryngeal surgery (with or without laser) for early laryngeal squamous cell cancer. Cochrane Database of Systematic Reviews, 2014(12), CD002027.

Willson, M. L., et al. (2019). Taxanes for adjuvant treatment of early breast cancer. Cochrane Database of Systematic Reviews, 2019(9), CD004421.

Xin, Y., et al. (2021). Immune checkpoint inhibitors plus neoadjuvant chemotherapy in early triple-negative breast cancer: A systematic review and meta-analysis. BMC Cancer, 21(1), 126.

Yusuf, S., et al. (1994). Effect of coronary artery bypass graft surgery on survival: Overview of 10-year results from randomised trials by the Coronary Artery Bypass Graft Surgery Trialists Collaboration. Lancet, 344(8922), 563–570.

Zhang, M., et al. (2022). Efficacy and safety of PD-1/PD-L1 inhibitors in triple-negative breast cancer: A systematic review and meta-analysis. Acta Oncologica, 61(9), 1105–1115.

**Table 2. Search strategies**

| **Breast cancer PICOs** | | | |
| --- | --- | --- | --- |
| CDSR (2023.01.09) | ‘breast cancer’ (ti/ab/kw) | 159 hits |  |
| Epistemonikos – only reviews on immunotherapy (2023.02.16) | In Title:  Filter: most recent 10 years  Filter: systematic reviews  ("breast cancer" OR "breast carcinoma" OR "mammary carcinoma") AND (ctla–4 OR pd–1 OR pd–l1 OR pd1 OR pdl1 OR checkpoint OR immunotherapy OR vaccine OR vaccination OR immune–modulation OR immunosuppression OR immunisation OR immunization) | 49 hits |  |
| **Laryngeal cancer, prostate cancer, and pancreatic cancer^a^ PICOs** | | | |
| CDSR (2023.03.01) | prostate cancer OR prostate carcinoma OR prostate neoplasm OR prostatic cancer OR prostatic carcinoma OR prostatic neoplasm (ti/ab/kw) | 50 hits |  |
|  | pancreas cancer OR pancreas carcinoma OR pancreas neoplasm OR pancreatic cancer OR pancreatic carcinoma OR pancreatic neoplasm OR pancreatic acinar carcinoma OR pancreatic acinar cell carcinoma OR pancreatic ductal adenocarcinoma OR pancreatic adenocarcinoma (ti/ab/kw) | 48 hits |  |
|  | Laryngeal cancer OR laryngeal carcinoma OR laryngeal neoplasm OR larynx cancer OR larynx carcinoma OR larynx neoplasm OR ‘laryngeal squamous cell cancer’ (ti/ab/kw) | 8 hits |  |
| Epistemonikos (2023.03.21) | In Title:  Filter: most recent 10 years  Filter: systematic reviews  (“prostate cancer” OR “prostate carcinoma” OR “prostate neoplasm” OR “prostatic cancer” OR “prostatic carcinoma” OR “prostatic neoplasm”  OR  “pancreas cancer” OR “pancreas carcinoma” OR “pancreas neoplasm” OR “pancreatic cancer” OR “pancreatic carcinoma” OR “pancreatic neoplasm” OR “pancreatic acinar carcinoma” OR “pancreatic acinar cell carcinoma” OR “pancreatic ductal adenocarcinoma” OR “pancreatic adenocarcinoma”)  AND  “locally advanced” OR T3 OR T4 | 165 hits (3 duplicates: 162 unique hits) |  |
|  | In Title:  Filter: most recent 10 years  Filter: systematic reviews  “Laryngeal cancer” OR “laryngeal carcinoma” OR “laryngeal neoplasm” OR “larynx cancer” OR “larynx carcinoma” OR “larynx neoplasm” OR “laryngeal squamous cell cancer” | 92 hits (5 duplicates: 87 unique) |  |
| **Prostate cancer and brachytherapy PICO (primary studies only)** | |  |  |
| Epistemonikos (2023.04.07) | In Title and Abstract  Filter: primary studies  (“prostate cancer” OR “prostate carcinoma” OR “prostate neoplasm” OR “prostatic cancer” OR “prostatic carcinoma” OR “prostatic neoplasm”)  AND  “brachytherapy”  AND  “locally advanced” OR T3 OR T4 | 43 hits |  |
| **Cardiology PICOs** | |  |  |
| CDSR (2023.04.14) | ("atrial fibrillation*" OR AF) AND (MAZE OR ablation* OR RFA) (ti/ab/kw) | 5 hits |  |
|  | pacemaker* (ti/ab/kw) | 7 hits (2 duplicates with atrial fibrillation; 5 unique) |  |
|  | "implantable cardioverter–defibrillator*" OR "implantable cardioverter defibrillator*" OR “implantable defibrillator*” OR ICD (ti/ab/kw) | 33 hits |  |
|  | “Acute coronary syndrome” OR infarct* OR “heart attack*” OR stemi OR nstemi OR angina OR “ischemic heart disease*” (ti/ab/kw)  AND  cabg or bypass OR “percutaneous coronary” OR pci OR angioplasty OR ptca (ti/ab/kw) | 53 hits |  |
| Epistemonikos (2023.04.21) | In Title and Abstract  Filter: systematic reviews^b^  ("implantable cardioverter–defibrillator*" OR "implantable cardioverter defibrillator*" OR “implantable defibrillator*” OR ICD) AND (“ventricular tachycardia” OR “ventricular fibrillation” OR “ventricular arrhythmias” OR VT OR VF) | 166 hits (2 duplicates: 164 unique) |  |
| Epistemonikos (2023.04.26) | In Title and Abstract  Filter: systematic reviews  ("atrial fibrillation*" OR AF) AND (MAZE OR “surgical ablation*”) | 61 hits (2 duplicates: 59 unique) |  |

Abbreviations. CDSR: Cochrane Database of Systematic Reviews

^a^ Pancreatic cancer was eventually excluded from the list of PICOs

^b^ It was decided not to restrict to reviews published in the most recent 10 years, as applying this restriction had little impact on the number of hits

## Quality of life (QoL) collection

**Methods and results of the Delphi study**

We carried out a two-round Delphi study to estimate the *QoL alternative treatment* and *QoL surgery* parameters. A Delphi study is a series of two or more survey rounds interspersed with controlled feedback (meaning that participants can see the results of the previous survey round) with the aim of achieving a consensus on a particular topic.

We aimed to recruit 30 physicians from diverse clinical fields, including from surgical and non-surgical specialties. A typical Delphi panel has 15-20 participants (1,2). Potential participants were non-randomly sampled from among personal contacts within and outside our affiliated institutions.

The first round of the Delphi study was designed as follows. First, we developed 30 clinical vignettes (see below), each corresponding to a specific combination of a disease and treatment such that all the PICOs were represented in the Delphi study. For multivessel diseases (CABG or PCI), we used previously obtained QoL data. Each vignette described the most prevalent symptoms associated with the disease and limitations imposed on daily life, with or without treatment. Each vignette concluded with the question: ‘how would you rate the following health state?’, to which participants could give an answer ranging from 0 to 100 using a visual analogue scale. For reference, participants were also provided with a visual analogue scale with reference QoL values, based on data from the Global Burden of Disease Study (3). Participants were encouraged to provide brief explanations for their estimates in free text fields.

To reduce the burden on the participants, we divided participants into two groups, each assigned about half of the vignettes. The surveys were administered using an online platform designed for conducting Delphi studies (4). After the first round, participants were invited for the second round that contained the same vignettes and questions. In this second round, participants were also presented with the results from the first round, including the medians and interquartile ranges of QoL life estimates across all participants for each vignette. Additionally, brief comments from four participants were included – two who assessed the quality of life as low and two who rated it as high to provoke reflection and possible regrading.

Quality of life estimates were re-analysed after the second round. The data of those who participated in round one but not in round two were excluded. 26 participants participated in round 1 (14 in group 1 and 12 in group 2). Twenty–five participants (96%) completed both Delphi rounds and were included in the analysis (n=13 in group 1 and n=12 in group 2). The median and interquartile ranges of quality of life estimates are shown below.

**Table 3. Summary of quality of life estimates (range 0 to 100) after the second round of the Delphi study (n=25)**

| **Panel 1 (n=13)** | **Median** | **Q25** | **Q75** |
| --- | --- | --- | --- |
| **Deafness** |  |  |  |
| Sign language (control) | 58 | 52 | 60 |
| Cochlear implant | 85 | 80 | 90 |
| Hearing aid | 70 | 69.25 | 72.75 |
| **Laryngeal carcinoma, T1–T2 M0** |  |  |  |
| No treatment (control) | 50 | 45 | 55 |
| Radiation therapy | 70 | 65 | 70 |
| Laser | 83 | 79 | 88.5 |
| **Laryngeal carcinoma, T3–T4** |  |  |  |
| No treatment (control) | 35 | 30 | 40 |
| Chemoradiation therapy | 60 | 55 | 64 |
| Radiation therapy | 61 | 55 | 70 |
| Total laryngectomy | 51 | 50 | 60 |
| **Benign prostatic hyperplasia** |  |  |  |
| Conservative treatment | 75 | 71 | 80 |
| Transurethral resection of prostate (TURP) | 85 | 80 | 87 |
| Laser | 90 | 90 | 95 |
| **Prostate cancer, M0** |  |  |  |
| No treatment (control) | 71 | 68 | 80 |
| Radical prostatectomy | 75 | 71 | 77 |
| External beam radiation | 80 | 75 | 80 |
| Brachytherapy | 84 | 79 | 87 |
| Hormone therapy | 80 | 75 | 80 |
| **Panel 2 (n=12)** | **Median** | **Q25** | **Q75** |
| **Bradycardia (due to sick sinus syndrome or 2^nd^ degree atrioventricular block)** |  |  |  |
| Medical therapy (control) | 67.5 | 62.75 | 71.25 |
| Pacemaker implantation | 90 | 89 | 90 |
| **Tachycardia (due to ventricular tachycardia or fibrillation)** |  |  |  |
| Medical therapy (control) | 70 | 60 | 74 |
| Implantable cardioverter–defibrillator (ICD) implantation | 82.5 | 79.75 | 86.25 |
| **Atrial fibrillation** |  |  |  |
| Medical therapy (control) | 78 | 71.5 | 81.25 |
| Ablation | 81 | 75 | 86 |
| MAZE procedure | 82.5 | 78.75 | 85 |
| **Breast cancer (M0, breast–conserving possible)** |  |  |  |
| No treatment (control) | 54.5 | 37.5 | 61.75 |
| Lumpectomy/breast–conserving surgery | 85 | 75 | 90 |
| Radiation therapy | 80 | 73.75 | 80 |
| Chemotherapy | 67.5 | 63.25 | 72 |
| Immunotherapy | 80 | 73.25 | 84 |
| **Breast cancer (M0, breast–conserving not possible)** |  |  |  |
| No treatment (control) | 42.5 | 33.75 | 51.25 |
| Mastectomy | 71.5 | 64 | 75 |
| **Knee osteoarthritis** |  |  |  |
| Conservative treatment (control) | 73 | 70 | 77 |
| Knee replacement | 86 | 85 | 90 |
| **Hip osteoarthritis** |  |  |  |
| Conservative treatment (control) | 71 | 69.5 | 76.25 |
| Hip replacement | 85 | 85 | 89.25 |

Abbreviations. Q25: first quartile; Q75: third quartile.

1. Akins, R. B., Tolson, H., & Cole, B. R. (2005). Stability of response characteristics of a Delphi panel: Application of bootstrap data expansion. BMC Medical Research Methodology, 5(1), 37.
2. Birko, S., Dove, E. S., & Özdemir, V. (2015). Evaluation of nine consensus indices in delphi foresight research and their dependency on delphi survey characteristics: A simulation study and debate on delphi design and interpretation. PLoS ONE, 10(8), e0135162.
3. Global Burden of Disease Collaborative Network. (2018). Global burden of disease study 2017 (GBD 2017) disability weights. Institute for Health Metrics and Evaluation (IHME).
4. Welphi. (2021). Welphi Application Internet. Retrieved from https:// www. welphi. com/ en/ Applications. html. Accessed 10 Apr 2023

**Table 4. Characteristics of the two expert panels which estimated the QoL weights**

| Speciality | Panel 1 | Panel 2 |
| --- | --- | --- |
| Surgical speciality | | |
| ENT surgeon not in training | 1 |  |
| General surgeon | 2 | 2 |
| Neurosurgeon |  | 1 |
| Plastic surgeon |  | 2 |
| Gynaecologist |  | 1 |
| Trauma surgeon | 1 |  |
| Orthopedic surgeon | 1 |  |
|  | 5 (38% of total) | 6 (50% of total) |
| Non–surgical speciality | | |
| Anaesthesiologist | 1 | 1 |
| General practitioner | 2 | 1 |
| Geriatrician | 2 |  |
| Internist | 1 | 1 |
| Rehabilitation medicine physician | 1 |  |
| Rehabilitation medicine physician in training |  | 1 |
| Pulmonologist |  | 1 |
| Psychiatrist | 1 | 1 |
|  |  |  |
| Total | 13 | 12 |

**Description of vignettes used in this study.**

1. **Deafness (paediatric population)**

Sign language: The children have severe sensorineural hearing loss. This condition is present from birth and is often detected during neonatal hearing screening. Severe hearing loss is generally experienced as a significant disability. Speech and language will not develop naturally in these children, and reduced language comprehension can later lead to learning difficulties and loneliness. Children can communicate with others using sign language and are thus part of the deaf culture. This culture is often considered valuable by deaf and hard-of-hearing individuals.

Cochlear implantation: A cochlear implant is implanted subcutaneously in the bone after a complete mastoidectomy. Cochlear implants generally yield the best results when inserted before the age of 1 year. Overall success with a cochlear implant depends on rehabilitation (which can take several years) with the help of speech therapy. Auditory functioning will be evaluated annually. More than 90% of the population addressed benefit from a cochlear implant. Both soft and loud sounds, as well as speech, can be perceived. However, the quality of sound and speech perception remains different to that of a normal auditory organ. About 6% experience complications, such as dizziness, taste problems, mastoiditis, or recurrent infections. In addition, certain sports like diving, kickboxing, and wrestling are discouraged.

Hearing aids: A hearing aid can be worn behind the ear or in the ear canal. It is important to fit and select appropriate hearing aids and ensure that the chosen hearing aid fits well. The hearing aid amplifies sound to an audible level. For children with severe to very severe hearing loss, a hearing aid will not provide sufficient compensation for the damaged inner ear to hear well. There is primarily discrimination loss: the maximum speech understanding, even in silence, is less than 100%. One issue with hearing aids is that they may not function well in situations with a lot of background noise.

1. **Laryngeal cancer T1-2, M0**

Radiation therapy: This treatment course lasts 5 to 7 weeks, with patients receiving a few minutes of radiation at least five times a week. Side effects of radiation can include a worsening of breathlessness or hoarseness, difficulty in eating, and local dermatitis. Any swallowing problems that do develop are generally mild and resolve themselves within 2-8 weeks of treatment. Long-term side effects are not uncommon for these patients and may include thick mucus, a dry throat, laryngeal oedema, chondritis, and larynx stenosis. The voice quality may also change. If there is a recurrence after treatment, radiation or laser treatment is no longer possible, making a total laryngectomy generally unavoidable.

Laser: Patients undergo laser surgery under general anaesthesia. Patients are not allowed to speak for three to five days after the surgery to give the vocal cords as much rest as possible. In the short term, patients may experience pain in the tongue, cheek, or lips. A risk from the surgery is damage to the teeth. Although voice quality is often poor immediately after treatment, it often improves later. If a recurrence occurs after treatment, radiation or renewed laser treatment is possible, reducing the likelihood of total laryngectomy.

1. **Laryngeal cancer T3-T4, M0**

Chemoradiation therapy: Generally healthy patients under 70 years old can be treated with chemoradiation therapy. This treatment takes place on a daily basis over six weeks. Possible side effects include dry mouth, thick mucus, dysphagia (with a possible dependence on tube feeding), aspiration, oesophageal strictures, and voice changes. Long-term side effects may include sensory disturbances, kidney function disorders, and/or hearing loss due to the chemotherapy.

Radiation therapy: Generally healthy patients over 70 years old can often be treated with radiation therapy. Patients with a T3 or very limited T4 tumour may also qualify for mono radiation therapy. This treatment lasts for six weeks and takes place on a daily basis. Possible side effects are dry mouth, thick mucus, dysphagia (with possible dependence on tube feeding), aspiration, oesophageal strictures, and voice changes.

Surgical resection (TLE): The total larynx is removed, and a tracheostoma is created during the surgery. After surgery, the patient stays in the hospital for several weeks. A voice prosthesis is inserted six weeks after the surgery. Approximately 30% of patients need a feeding tube due to aspiration or eating problems. The main advantage of this surgery is that patients can breathe normally. The disadvantage is that patients lose their voice and need to learn to speak using the voice prosthesis. Total laryngectomy is a complicated surgery with considerable risks, including damage to the carotid arteries and thrombosis. After the surgery, patients often suffer from mucus retention. The tumour will often have infiltrated the strap muscles. If the cancer recurs, the treatment is very difficult, and the prognosis is poor. Radiation therapy and/or chemotherapy are often no longer possible.

1. **Symptomatic bradycardia (due to sick sinus syndrome, second-degree AV block)**

Optimal medical therapy: Patients with cardiac arrhythmias have a slow heart rate that can lead to a general feeling of discomfort, dizziness, fatigue, a tendency to faint, and sometimes chest pain during exertion. This can be limiting for the patient in their daily life. In an acute episode where patients become severely bradycardic, leading to syncope, shock, or myocardial ischemia, patients should be treated with medication by a cardiologist (e.g., atropine). Additionally, there is a risk that the patient may die from severe bradycardia, which can cause anxiety.

Pacemaker implantation: During this procedure, a pacemaker is implanted just under the skin or chest muscle. Possible complications of this procedure include pacemaker displacement (1-5%), which may require a new procedure, bleeding (0.5-3%), infection (0.9-3%), pneumothorax (0.6-3%), and cardiac tamponade (0.1-4%). After an implantation, patients need to come in for regular check-ups. In most cases, no limitations are placed on their daily lives. Pacemakers operate on a battery and typically last 5-15 years.

1. **Ventricular arrhythmias**

Optimal medical therapy: A ventricular tachycardia can manifest itself through symptoms such as palpitations, sweating, an uncomfortable feeling, nausea, dizziness, and syncope. Further, it can transition into ventricular fibrillation, a life-threatening situation where blood circulation stops, leading to cardiac arrest. The risk of ventricular fibrillation can cause anxiety. Ventricular tachycardia can be treated with anti-arrhythmic drugs, with beta-blockers, which are effective and generally safe, being the first choice. Side effects associated with beta-blockers may include fatigue, gastrointestinal disturbances, depression, dizziness, and headaches.

ICD: An implantable cardioverter-defibrillator (ICD) can be inserted to prevent the severe consequences of ventricular tachycardia. In the event of an episode, the ICD delivers electrical impulses to neutralize the abnormal heart rhythms in the ventricles, or provides a shock to restore the heart to its normal rhythm. The ICD is implanted under the skin near the collarbone and has leads that travel through blood vessels to the heart. Risks during implantation include pneumothorax (collapsed lung), hiccups, or perforation of the heart wall. After implantation, the risks may include bleeding, infection, dislodged leads, or inappropriate shocks. The average lifespan of an ICD is 6-8 years.

1. **Persistent atrial fibrillation**

Optimal medical therapy: Patients with persistent and symptomatic atrial fibrillation may experience palpitations, shortness of breath, dizziness, and angina pectoris. They have an increased risk of thromboembolic complications (TIA, stroke, peripheral thromboembolism). Hemodynamically stable patients can receive long-term medical treatment using beta-blockers, calcium channel blockers, or digoxin. In addition, patients are prescribed anticoagulants to prevent thromboembolic complications.

Cardiac ablation: This procedure is performed under local anaesthesia and intravenous pain relief. Catheters are introduced into the heart through the groin, creating scars to block the electrical signals. Possible complications include groin bleeding or swelling, the formation of blood clots and possible stroke, narrowing of a pulmonary vein, or damage to the phrenic nerve. After discharge, patients should avoid straining the groin area for a week. Approximately 70-75% of patients are symptom-free one year after the treatment.

Maze procedure: During this procedure, the patient is placed under general anaesthesia and connected to a heart-lung machine. Scar tissue is created to prevent incorrect electrical signals from reaching the ventricles. It is a relatively safe procedure, but possible complications include infection, postoperative bleeding, or the recurrence of arrhythmias. Patients often spend a night in the intensive care unit (ICU) after surgery. It may take three to six months for the definitive results of the procedure to be clear. Consequently, patients continue taking the same medications as before during this initial period. In the long term, most patients achieve a regular heart rhythm.

1. **Breast cancer, T1-2 M0**

Radiation therapy: Patients receiving radiation therapy start with a planning session to map the tumour in relation to surrounding structures. Depending on the chosen type of radiation therapy, there may be one or two radiation sessions per day, with a maximum therapy duration of 5-5.5 weeks. Radiation can also be applied through brachytherapy (placement of radioactive material in the body) or during surgery. Significant long-term side effects of radiation therapy include second malignancies (near the radiation field), pneumonitis, lung fibrosis, heart damage, and lymphedema.

Chemotherapy: Chemotherapy for breast cancer is given in multiple cycles, with their duration and quantity depending on the chosen regimen. The cycle duration is about 14-28 days, and there are 4-8 cycles. Short-term side effects include infection, dehydration, nausea, anaemia, and malnutrition. Long-term side effects of systemic therapy depend on the type of chemotherapeutic agent and may include second malignancies (e.g., leukaemia), ototoxicity, cardiomyopathy, kidney toxicity, early menopause and infertility, sexual dysfunction, osteoporosis, neuropathy, cognitive dysfunction, weight gain, and fatigue.

Chemotherapy plus immunotherapy: Patients are often treated with immunotherapy for at least one year, and this treatment is often combined with chemotherapy. Various medications can be administered (via infusion, injection, or in tablet form). One commonly administered drug (trastuzumab) is given once every three weeks by infusion or subcutaneous injection. Side effects are usually mild, such as flu-like symptoms, fatigue, or gastrointestinal complaints. A significant but less common side effect is a deterioration in heart function. Therefore, regular echocardiograms are performed, and provided the therapy is stopped in a timely manner, full recovery usually occurs.

Lumpectomy/breast-conserving surgery: During the procedure, a sentinel lymph node procedure is often performed with the possibility of an axillary lymph node dissection. If a significant portion of the breast needs to be removed, a plastic surgeon may assist with reconstruction. Complications may include seroma (8.4%), bleeding, infection (1-5%), and thrombosis (0.16%). After the surgery, the breast may be somewhat smaller and have a different shape. The nipple may also be slightly displaced, and there may be reduced sensation around the scar. Usually, patients can fully function as they did before the surgery within 1-2 weeks of the surgery.

1. **Breast cancer T3-4 M0**

Mastectomy: A mastectomy is performed when the breast cancer is too widespread for breast-conserving surgery or as a preventive measure in cases of hereditary breast cancer. During the procedure, the entire breast gland, nipple, and lymph nodes in the armpit are removed. Depending on the size of the tumour, the pectoralis major muscle may also need to be removed. Sometimes, breast reconstruction is performed immediately, during the amputation. Complications may include chronic pain (10%), lymphedema (3.5-20%), postoperative bleeding, infection (1-5%), nipple necrosis (5.9%), and thrombosis (0.16%). After such surgery, patients should avoid heavy physical activity for 4-6 weeks. If an axillary lymph node dissection is performed, it can lead to reduced lymph drainage, potentially causing bothersome lymphedema in the arm.

For the alternative treatments in case of a mastectomy (i.e., Chemotherapy + Immunotherapy, Chemotherapy, Radiation therapy) no new vignettes were established to obtain QoL estimates. The estimates obtained from the vignettes belonging to lumpectomy were used.

1. **Benign prostatic hyperplasia (BPH)**

Conservative, medication with transurethral catheter: This condition commonly affects older men (80% of men above the age of 80 are affected). Patients experience symptoms such as a weaker urinary stream, dribbling, urinary incontinence, polyuria, nocturia, severe urgency symptoms, and sometimes bladder retention leading to urinary tract infections. Symptoms generally worsen over time. Medical treatment can involve the use of alpha-blockers or 5-alpha-reductase inhibitors. However, the effectiveness of both is limited. With the use of alpha-blockers, side effects such as a general malaise, headaches, and gastrointestinal issues may occur in 1-10% of patients. The use of 5-alpha-reductase inhibitors may lead to erectile dysfunction and/or reduced libido in 1-10% of patients.

Transurethral Resection of the Prostate (TURP): TURP aims to remove or make more accessible an obstructing prostate (either presumed or proven through urodynamic testing). The primary complications of this treatment are incontinence (7%) and postoperative urinary retention due to urethral stricture (8%). The urethral wound generally heals within 6-8 weeks, allowing patients to resume all their daily activities. Incontinence problems typically subside once the surgical site is healed. Some patients may continue to experience issues with incontinence (7%), erectile dysfunction, and ejaculation disorders. Bladder neck contractures and urethral strictures may also develop.

Laser therapy: In this treatment, a laser fibre is inserted through the urethra via a thin working shaft (thinner than in traditional surgery) to vaporize excess prostate tissue. This procedure has a lower risk of damaging the urethra. There are minimal bleeding incidents during or after the treatment, and a catheter is only needed for a short duration (24 hours). Patients recover quickly from this treatment. Once urination is re-established after catheter removal, the patient can return home. Most patients experience a significant improvement in urinary symptoms within 24 hours. However, for a few weeks, patients may still experience urgency, bladder spasms, and a slight burning sensation during urination; these symptoms usually resolve themselves over time.

1. **Locally advanced prostate cancer**

External beam radiation: This treatment typically lasts 8 weeks, with short daily radiation sessions (up to 5 minutes) administered 3 to 5 days a week. Patients do not need to be hospitalized for this. During and after radiation, patients may experience fatigue, local skin irritation, bowel issues, erectile problems, and urinary symptoms (burning sensation, haematuria). One year after radiation, around 1 in 10 men still experience significant side effects.

Hormone therapy: This treatment is often used in addition to radiation therapy. Patients receive injections every 3 to 6 months, leading to the cessation of testosterone production (often referred to as chemical castration). Common side effects include changes in appearance (reduced muscle mass, less body hair), sexual problems, and symptoms resembling menopausal effects (hot flushes, fatigue, osteoporosis). The treatment can be temporarily paused in the event of severe side effects, which does not affect life expectancy.

Prostatectomy: Complications that may occur during or shortly after this procedure affect less than 10% of patients and include postoperative bleeding, wound infection, urethral stricture (8%), bowel perforation, or lymphedema in the lower abdomen, thighs, and/or scrotum. Patients can typically be discharged within 4-5 days. After recovery, some patients may experience lasting side effects of the procedure. Incontinence symptoms usually resolve themselves, but 1 in 14 men can still experience significant issues one year after the surgery. If the procedure was performed with nerve-sparing and there were no preoperative erectile problems, there is approximately a 60% chance of recovery after the surgery. A retrograde ejaculation may occur during orgasm. If incontinence or erectile dysfunction persists, this can significantly impact the quality of life.

1. **Knee osteoarthritis**

Conservative treatment: Patients are typically over 60-65 years of age. They often experience increasingly severe symptoms, such as knee pain and pain in the back of the knee with radiating discomfort in the lower leg. This leads to mobility limitations, especially during flexion. The pain increases after physical activity, and there is morning stiffness. Possible complications include infection (1-2%), thrombosis (0.2-1%), and nerve damage (0.79%) that can result in foot drop. Symptomatic treatment options include pain management, regular physical activity, physical therapy, braces, and other supportive devices.

Knee replacement: After such surgery, the pain gradually decreases. The rehabilitation period is extensive, lasting up to one year post surgery. Most patients have fully recovered after one year. The artificial knee prosthesis may have limited flexion compared to a natural knee. Patients can often resume sports activities, although this might be less intensive than before, and some sports may not be appropriate. Staying physically active and maintaining a healthy weight are crucial. An artificial knee typically lasts for 15 to 20 years, after which revision surgery may be necessary.

1. **Hip osteoarthritis**

Conservative treatment: Patients are typically over 60-65 years of age, most often female, and experience severe symptoms such as groin pain radiating to the upper leg. They often have difficulty in initiating movement and stiffness, as well as limitations in hip joint function, especially during flexion and weaknesses during abduction. Symptomatic treatment options include pain management, regular physical activity, physical therapy, braces, and other supportive devices.

Hip replacement: Pain typically decreases immediately after this surgery. Most patients remain hospitalized for 2 to 5 days following the procedure. Potential complications during hip replacement may include femoral or pelvic fractures (1-18%), infection (0.4-2.2%), leg length discrepancy (1-27%), nerve damage (1-2%) leading to reduced sensation and conditions such as foot drop (recovery is often complete within one year). Rehabilitation typically begins within hours of the surgery and may take weeks or months, usually resulting in good hip function. An artificial hip can last for more than 20 years. In some cases, hip dislocation may occur, usually within three months, requiring repositioning in an emergency room or operating theatre.

The following vignettes were used in our previous study to estimate QoL. These estimates were subsequently incorporated into the present study. Nonetheless, for a comprehensive understanding of the vignettes employed, these will be outlined below.

1. **Multivessel disease**

Optimal medical therapy: It concerns patients (mostly > 50 years old) with a long-standing elevated cardiovascular risk profile. Patients present with exertion-related chest pain: the chest pain diminishes at rest and/or with sublingual nitrates.

CABG: It involves open-heart surgery. Often, an artery is removed through an incision of 30-60 cm in the lower leg to use as a bypass. Possible complications include: heart attack during surgery (2-4%), postoperative bleeding, cardiac arrhythmias, and infections of the pericardium, sternum (0.9-1.3%), or wounds (1-24%). Patients generally recover well from this surgery in about 3 months. However, their significantly elevated cardiovascular risk profile persists and impacts their well-being and confidence in their own bodies. Patients must use antiplatelet drugs, cholesterol-lowering medications, and beta-blockers for life.

PCI: A catheter is inserted into an artery in the groin, wrist, or elbow. The narrowing in the coronary arteries is alleviated through balloon dilation. In most cases, a stent is immediately placed to keep the coronary artery open. Severe complications such as the occurrence of cardiac arrhythmias, arterial wall tear (0.2-0.6%), or the formation of a clot leading to a heart or brain infarction (0.5-1%) are rare. After the procedure, patients recover quickly and are usually relieved of their symptoms. However, their significantly elevated cardiovascular risk profile persists and has an impact on their well-being and confidence in their own bodies. Patients must take antiplatelet drugs and heart medication for life.

**Visual analogue scale (VAS) used to collect the QoL data**


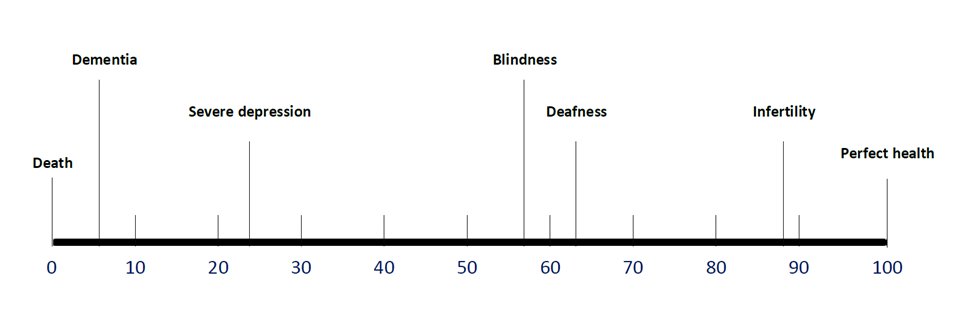
Five available QoL estimates (for dementia, severe depression, blindness, deafness, and infertility) from the World Health Organization Global Burden of Disease study were made available to provide reference points.

## Supplementary material 3

References used in table 3 of the manuscript:

1. Yusuf, S., et al., Effect of coronary artery bypass graft surgery on survival: overview of 10–year results from randomised trials by the Coronary Artery Bypass Graft Surgery Trialists Collaboration. Lancet, 1994. 344(8922): p. 563–70.

2. Bakhai, A., et al., Percutaneous transluminal coronary angioplasty with stents versus coronary artery bypass grafting for people with stable angina or acute coronary syndromes. Cochrane Database Syst Rev, 2005. 2005(1): p. CD004588.

3. Bruijnzeel, H., et al., Evaluation of pediatric cochlear implant care throughout Europe: Is European pediatric cochlear implant care performed according to guidelines? Cochlear Implants Int, 2017. 18(6): p. 287–296.

4. Klop, C., et al., COX–2–selective NSAIDs and risk of hip or knee replacements: a population–based case–control study. Calcif Tissue Int, 2012. 91(6): p. 387–94.

5. Ozaydin, M., et al., Long–Term Outcome of Patients With Idiopathic Ventricular Fibrillation: A Meta–Analysis. J Cardiovasc Electrophysiol, 2015. 26(10): p. 1095–104.

6. Parkes, J., J. Bryant, and R. Milne, Implantable cardioverter–defibrillators in arrhythmias: a rapid and systematic review of effectiveness. Heart, 2002. 87(5): p. 438–42.

7. van der Lingen, A.C.J., et al., Recurrent ventricular arrhythmias and mortality in cardiac arrest survivors with a reversible cause with and without an implantable cardioverter defibrillator: A systematic review. Resuscitation, 2022. 173: p. 76–90.

8. Nanda, A., et al., Oncoplastic breast–conserving surgery for women with primary breast cancer. Cochrane Database Syst Rev, 2021. 10(10): p. CD013658.

9. Kindts, I., et al., Tumour bed boost radiotherapy for women after breast–conserving surgery. Cochrane Database Syst Rev, 2017. 11(11): p. CD011987.

10. Li, Y., et al., Efficacy and Safety of Adding Immune Checkpoint Inhibitors to Neoadjuvant Chemotherapy Against Triple–Negative Breast Cancer: A Meta–Analysis of Randomized Controlled Trials. Front Oncol, 2021. 11: p. 657634.

11. Mittal, N., et al., Immune checkpoint inhibitors as neoadjuvant therapy in early triple–negative breast cancer: A systematic review and meta–analysis. J Cancer Res Ther, 2022. 18(6): p. 1754–1765.

12. Sternschuss, M., et al., Efficacy and safety of neoadjuvant immune checkpoint inhibitors in early–stage triple–negative breast cancer: a systematic review and meta–analysis. J Cancer Res Clin Oncol, 2021. 147(11): p. 3369–3379.

13. Xin, Y., et al., Immune checkpoint inhibitors plus neoadjuvant chemotherapy in early triple–negative breast cancer: a systematic review and meta–analysis. BMC Cancer, 2021. 21(1): p. 1261.

14. Zhang, M., et al., Efficacy and safety of PD–1/PD–L1 inhibitors in triple–negative breast cancer: a systematic review and meta–analysis. Acta Oncol, 2022. 61(9): p. 1105–1115.

15. Willson, M.L., et al., Taxanes for adjuvant treatment of early breast cancer. Cochrane Database Syst Rev, 2019. 9(9): p. CD004421.

16. Bui, K.T., et al., Ovarian suppression for adjuvant treatment of hormone receptor–positive early breast cancer. Cochrane Database Syst Rev, 2020. 3(3): p. CD013538.

17. Hoon, S.N., et al., Capecitabine for hormone receptor–positive versus hormone receptor–negative breast cancer. Cochrane Database Syst Rev, 2021. 5(5): p. CD011220.

18. Hickey, B.E. and M. Lehman, Partial breast irradiation versus whole breast radiotherapy for early breast cancer. Cochrane Database Syst Rev, 2021. 8(8): p. CD007077.

19. Mota, B.S., et al., Nipple– and areola–sparing mastectomy for the treatment of breast cancer. Cochrane Database Syst Rev, 2016. 11(11): p. CD008932.

20. Barnett, S.D. and N. Ad, Surgical ablation as treatment for the elimination of atrial fibrillation: a meta–analysis. J Thorac Cardiovasc Surg, 2006. 131(5): p. 1029–35.

21. Berger, W.R., et al., Persistent atrial fibrillation: A systematic review and meta–analysis of invasive strategies. Int J Cardiol, 2019. 278: p. 137–143.

22. Nyong, J., et al., Efficacy and safety of ablation for people with non–paroxysmal atrial fibrillation. Cochrane Database Syst Rev, 2016. 11(11): p. CD012088.

23. Doyle, J.F. and K.M. Ho, Benefits and risks of long–term amiodarone therapy for persistent atrial fibrillation: a meta–analysis. Mayo Clin Proc, 2009. 84(3): p. 234–42.

24. Dretzke, J., et al., Dual chamber versus single chamber ventricular pacemakers for sick sinus syndrome and atrioventricular block. Cochrane Database Syst Rev, 2004. 2004(2): p. CD003710.

25. Shaw, D.B., et al., Survival in second degree atrioventricular block. Br Heart J, 1985. 53(6): p. 587–93.

26. Marra, G., et al., Management of Patients with Node–positive Prostate Cancer at Radical Prostatectomy and Pelvic Lymph Node Dissection: A Systematic Review. Eur Urol Oncol, 2020. 3(5): p. 565–581.

27. Fahmy, O., et al., The Role of Radical Prostatectomy and Radiotherapy in Treatment of Locally Advanced Prostate Cancer: A Systematic Review and Meta–Analysis. Urol Int, 2017. 99(3): p. 249–256.

28. Lei, J.H., et al., Androgen–deprivation therapy alone versus combined with radiation therapy or chemotherapy for nonlocalized prostate cancer: a systematic review and meta–analysis. Asian J Androl, 2016. 18(1): p. 102–7.

29. Dong, Z., et al., Intermittent hormone therapy versus continuous hormone therapy for locally advanced prostate cancer: a meta–analysis. Aging Male, 2015. 18(4): p. 233–7.

30. Warner, L., et al., Radiotherapy versus open surgery versus endolaryngeal surgery (with or without laser) for early laryngeal squamous cell cancer. Cochrane Database Syst Rev, 2014. 2014(12): p. CD002027.

31. Pedregal–Mallo, D., et al., Oncological and functional outcomes of transoral laser surgery for laryngeal carcinoma. Eur Arch Otorhinolaryngol, 2018. 275(8): p. 2071–2077.

32. Francis, E., et al., T4a laryngeal cancer survival: retrospective institutional analysis and systematic review. Laryngoscope, 2014. 124(7): p. 1618–23.

33. Fu, X., Q. Zhou, and X. Zhang, Efficacy Comparison Between Total Laryngectomy and Nonsurgical Organ–Preservation Modalities in Treatment of Advanced Stage Laryngeal Cancer: A Meta–Analysis. Medicine (Baltimore), 2016. 95(14): p. e3142.

## Table 1. The model outcome for all “disease–surgery–alternative treatment” combinations. The estimates and 95% confidence intervals are shown. These outcomes were determined through 1000 probabilistic sensitivity analysis (PSA) iterations. The model outcome for a 2-week surgical delay was considered direct surgery, and served as the reference value in this case.

| DALYs of alternative treatment | | | | | | | |
| --- | --- | --- | --- | --- | --- | --- | --- |
|  | 12 weeks | 22 weeks | 32 weeks | 42 weeks | 52 weeks | Lifelong | Per month |
| Breast cancer, T1–2: Chemotherapy + Immunotherapy vs. Lumpectomy | 0.59 (0.46 - 0.71) | 1.15 (0.89 - 1.39) | 1.68 (1.30 - 2.03) | 2.18 (1.69 - 2.63) | 2.66 (2.07 - 3.20) | 11.60 (9.32 - 13.51) | 0.23 (0.18 - 0.28) |
| Breast cancer, T3–4: Chemotherapy + Immunotherapy vs. Mastectomy | 0.50 (0.36 - 0.62) | 0.97 (0.70 - 1.21) | 1.41 (1.03 - 1.76) | 1.84 (1.34 - 2.29) | 2.24 (1.64 - 2.79) | 9.88 (7.42 - 12.09) | 0.19 (0.14 - 0.24) |
| Symptomatic bradycardia: Optimal medical therapy vs. Pacemaker implantation | 0.35 (0.21 - 0.51) | 0.67 (0.40 - 0.98) | 0.98 (0.59 - 1.43) | 1.28 (0.76 - 1.85) | 1.55 (0.92 - 2.24) | 6.21 (3.22 - 8.72) | 0.13 (0.08 - 0.19) |
| Ventricular arrhythmias: Optimal medical therapy vs. ICD | 0.19 (0.14 - 0.24) | 0.37 (0.28 - 0.47) | 0.55 (0.41 - 0.69) | 0.72 (0.53 - 0.91) | 0.88 (0.65 - 1.11) | 5.09 (3.33 - 6.67) | 0.08 (0.06 - 0.10) |
| Breast cancer, T1–2: Chemotherapy vs. Lumpectomy | 0.09 (0.04 - 0.16) | 0.18 (0.07 - 0.31) | 0.26 (0.11 - 0.47) | 0.35 (0.14 - 0.62) | 0.43 (0.18 - 0.77) | 5.45 (2.33 - 8.32) | 0.04 (0.02 - 0.07) |
| Deafness (pediatric population): Sign language vs. Cochlear implantation | 0.05 (0.03 - 0.08) | 0.11 (0.06 - 0.16) | 0.16 (0.09 - 0.25) | 0.22 (0.12 - 0.33) | 0.27 (0.15 - 0.41) | 13.06 (6.93 - 19.60) | 0.02 (0.01 - 0.04) |
| Breast cancer, T3–4: Chemotherapy vs. Mastectomy | 0.05 (0.00 - 0.12) | 0.11 (0.01 - 0.23) | 0.16 (0.01 - 0.34) | 0.22 (0.02 - 0.45) | 0.27 (0.02 - 0.56) | 3.43 (0.03 - 6.56) | 0.02 (0.00 - 0.05) |
| Multivessel disease: Optimal medical therapy vs. CABG | 0.04 (0.01 - 0.08) | 0.08 (0.01 - 0.15) | 0.11 (0.02 - 0.23) | 0.15 (0.02 - 0.30) | 0.19 (0.03 - 0.37) | 2.12 (0.30 - 4.47) | 0.02 (0.00 - 0.03) |
| Breast cancer, T1–2: Radiation therapy vs. Lumpectomy | 0.04 (0.01 - 0.07) | 0.08 (0.02 - 0.13) | 0.11 (0.03 - 0.19) | 0.15 (0.04 - 0.25) | 0.18 (0.05 - 0.32) | 1.27 (0.10 - 2.43) | 0.02 (0.00 - 0.03) |
| Locally advanced prostate cancer: Hormone therapy vs. Prostatectomy | 0.03 (0.00 - 0.06) | 0.07 (0.01 - 0.12) | 0.10 (0.01 - 0.18) | 0.14 (0.02 - 0.24) | 0.17 (0.02 - 0.30) | 2.32 (-0.11 - 4.50) | 0.01 (0.00 - 0.03) |
| Deafness (pediatric population): Hearing aids vs. Cochlear implantation | 0.03 (0.01 - 0.05) | 0.06 (0.02 - 0.09) | 0.09 (0.03 - 0.14) | 0.11 (0.04 - 0.18) | 0.14 (0.05 - 0.23) | 6.86 (2.52 - 10.78) | 0.01 (0.00 - 0.02) |
| Hip osteoarthritis: Conservative vs. Hip replacement | 0.03 (0.01 - 0.04) | 0.05 (0.02 - 0.09) | 0.08 (0.03 - 0.13) | 0.11 (0.04 - 0.18) | 0.13 (0.05 - 0.22) | 1.94 (0.71 - 3.83) | 0.01 (0.00 - 0.02) |
| Knee osteoarthritis: Conservative vs. Knee replacement | 0.03 (0.01 - 0.05) | 0.05 (0.01 - 0.09) | 0.08 (0.02 - 0.14) | 0.10 (0.03 - 0.19) | 0.13 (0.03 - 0.23) | 2.00 (0.49 - 3.66) | 0.01 (0.00 - 0.02) |
| Benign prostatic hyperplasia: Conservative vs. TURP | 0.02 (-0.01 - 0.05) | 0.04 (-0.03 - 0.10) | 0.05 (-0.04 - 0.16) | 0.07 (-0.06 - 0.20) | 0.09 (-0.07 - 0.25) | 0.59 (-0.90 - 1.98) | 0.01 (-0.01 - 0.02) |
| Locally advanced prostate cancer: External beam radiation vs. Prostatectomy | 0.01 (-0.00 - 0.03) | 0.03 (-0.00 - 0.05) | 0.04 (-0.01 - 0.08) | 0.05 (-0.01 - 0.10) | 0.07 (-0.01 - 0.13) | 0.91 (-0.13 - 1.78) | 0.01 (-0.00 - 0.01) |
| Persistent AF: Optimal medical therapy vs. Maze procedure | 0.01 (-0.03 - 0.05) | 0.02 (-0.05 - 0.10) | 0.03 (-0.08 - 0.15) | 0.05 (-0.11 - 0.20) | 0.06 (-0.13 - 0.25) | 0.92 (-2.18 - 3.87) | 0.00 (-0.01 - 0.02) |
| Breast cancer, T3–4: Radiation therapy vs. Mastectomy | 0.00 (-0.01 - 0.01) | 0.00 (-0.02 - 0.03) | 0.00 (-0.04 - 0.04) | 0.00 (-0.05 - 0.05) | 0.00 (-0.06 - 0.07) | -0.25 (-1.33 - 0.77) | 0.00 (-0.01 - 0.01) |
| Persistent AF: Cardiac ablation vs. Maze procedure | -0.01 (-0.04 - 0.03) | -0.01 (-0.09 - 0.06) | -0.02 (-0.13 - 0.09) | -0.03 (-0.17 - 0.12) | -0.04 (-0.22 - 0.15) | -0.73 (-3.77 - 2.14) | -0.00 (-0.02 - 0.01) |
| Benign prostatic hyperplasia: Laser therapy vs. TURP | -0.01 (-0.03 - 0.01) | -0.02 (-0.05 - 0.02) | -0.03 (-0.08 - 0.02) | -0.04 (-0.10 - 0.03) | -0.05 (-0.13 - 0.04) | -0.66 (-1.76 - 0.57) | -0.00 (-0.01 - 0.00) |
| Multivessel disease: PCI vs. CABG | -0.01 (-0.04 - 0.01) | -0.02 (-0.07 - 0.02) | -0.04 (-0.11 - 0.03) | -0.05 (-0.14 - 0.04) | -0.06 (-0.17 - 0.05) | -0.59 (-2.05 - 0.52) | -0.01 (-0.02 - 0.00) |
| Laryngeal cancer T3–4: Radiation therapy vs. Surgical resection (TLE) | -0.02 (-0.06 - 0.03) | -0.03 (-0.11 - 0.05) | -0.05 (-0.17 - 0.08) | -0.06 (-0.22 - 0.10) | -0.07 (-0.27 - 0.13) | -0.39 (-1.47 - 0.60) | -0.01 (-0.02 - 0.01) |
| Laryngeal cancer T3–4: Chemoradiation vs. Surgical resection (TLE) | -0.02 (-0.05 - 0.00) | -0.04 (-0.09 - 0.00) | -0.06 (-0.13 - 0.01) | -0.08 (-0.18 - 0.01) | -0.10 (-0.22 - 0.01) | -0.79 (-3.09 - 1.41) | -0.01 (-0.02 - 0.00) |
| Laryngeal cancer T1–2: Radiation therapy vs. Surgical resection (laser) | -0.02 (-0.06 - 0.01) | -0.04 (-0.11 - 0.02) | -0.07 (-0.17 - 0.03) | -0.09 (-0.22 - 0.05) | -0.11 (-0.27 - 0.06) | -0.62 (-1.65 - 0.29) | -0.01 (-0.02 - 0.00) |

AF: atrial fibrillation, CABG: coronary artery bypass graft, ICD: implantable cardioverter–defibrillator, PCI: percutaneous coronary intervention, TLE: total laryngectomy, TURP: transurethral resection of the prostate.

## The model outcome for all 23 disease–surgery–treatment combinations.

This outcome can be shown in two modes: 1) per month of alternative treatment and 2) lifelong alternative treatment.

**Figure 1. Per month of alternative treatment**


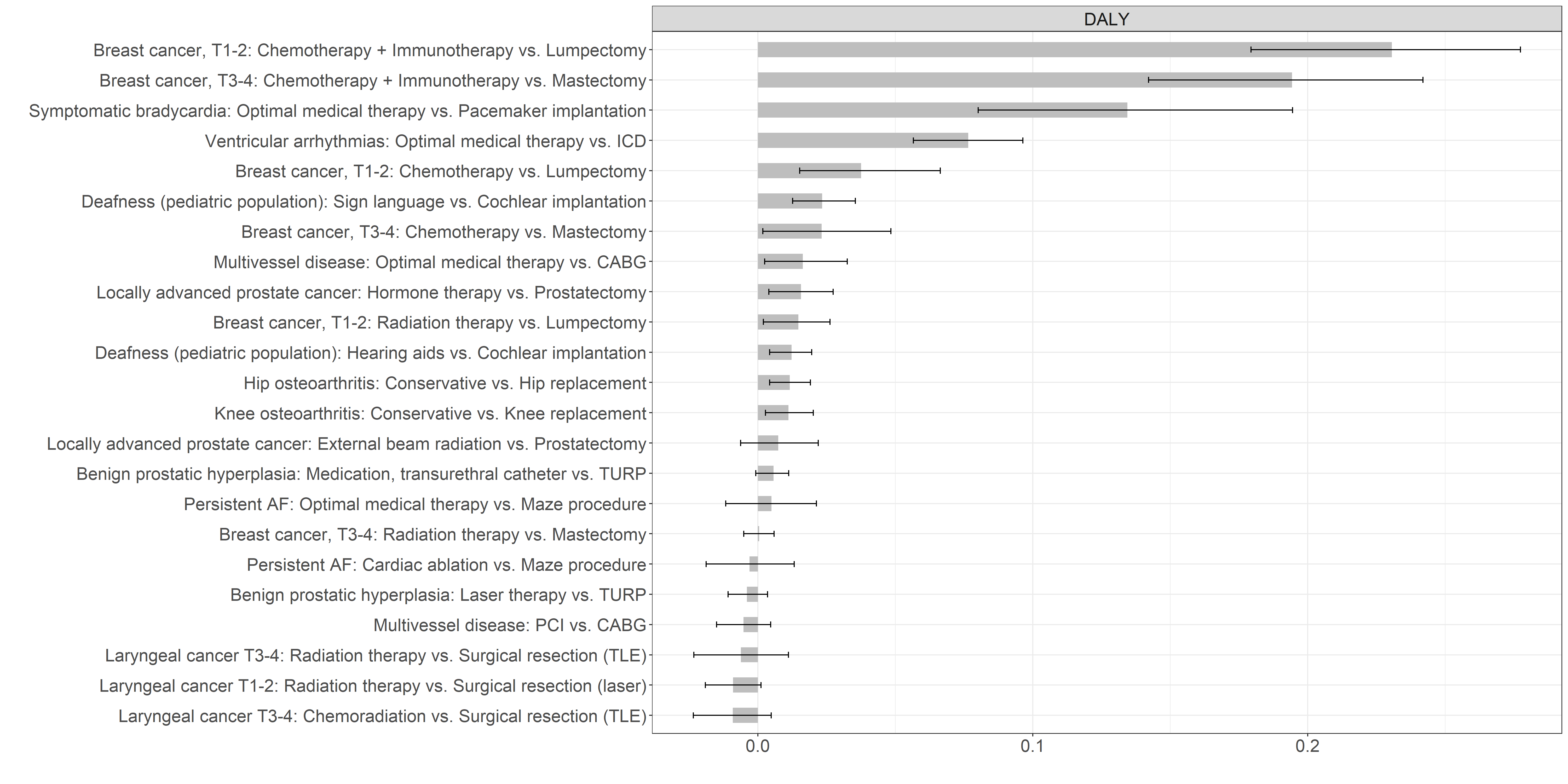


AF: atrial fibrillation, CABG: coronary artery bypass graft, ICD: implantable cardioverter–defibrillator, PCI: percutaneous coronary intervention, TLE: total laryngectomy, TURP: transurethral resection of the prostate.

**Figure 2. Lifelong alternative treatment**


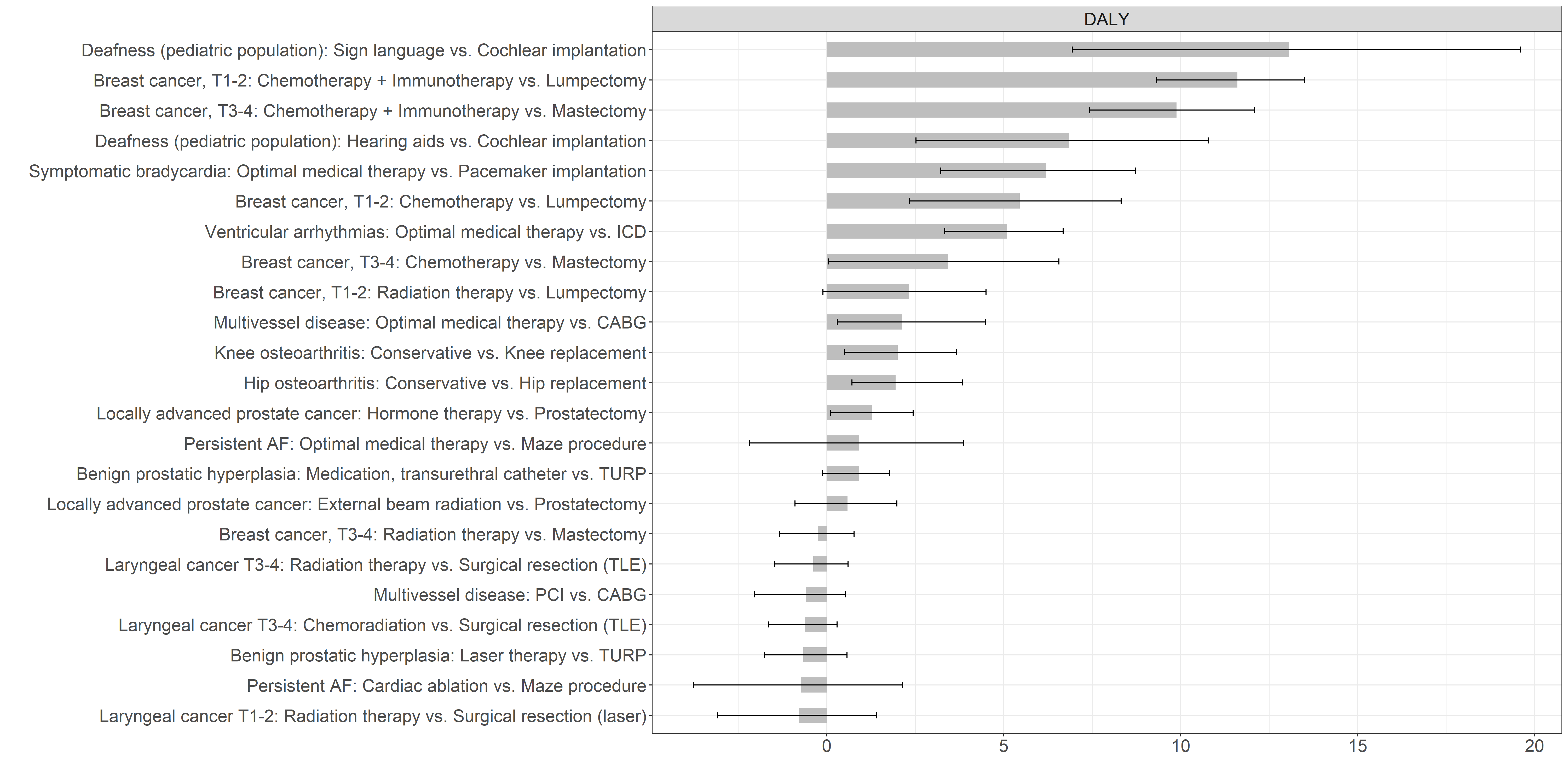


AF: atrial fibrillation, CABG: coronary artery bypass graft, ICD: implantable cardioverter–defibrillator, PCI: percutaneous coronary intervention, TLE: total laryngectomy, TURP: transurethral resection of the prostate.
